# Evolution-proof inhibitors of public good cooperation: a screening strategy inspired by social evolution theory

**DOI:** 10.1093/femsre/fuac019

**Published:** 2022-06-08

**Authors:** Maries Lissens, Mathieu Joos, Bram Lories, Hans P Steenackers

**Affiliations:** Centre of Microbial and Plant Genetics (CMPG), Department of Microbial and Molecular Systems, KU Leuven, Leuven, B-3001, Belgium; Centre of Microbial and Plant Genetics (CMPG), Department of Microbial and Molecular Systems, KU Leuven, Leuven, B-3001, Belgium; Centre of Microbial and Plant Genetics (CMPG), Department of Microbial and Molecular Systems, KU Leuven, Leuven, B-3001, Belgium; Centre of Microbial and Plant Genetics (CMPG), Department of Microbial and Molecular Systems, KU Leuven, Leuven, B-3001, Belgium

**Keywords:** Anti-virulence drugs, Antimicrobial resistance, Microbial social evolution, Evolutionarily-robust drugs, Drug screening/target identification, Interference with public good cooperation

## Abstract

Interference with public good cooperation provides a promising novel antimicrobial strategy since social evolution theory predicts that resistant mutants will be counter-selected if they share the public benefits of their resistance with sensitive cells in the population. Although this hypothesis is supported by a limited number of pioneering studies, an extensive body of more fundamental work on social evolution describes a multitude of mechanisms and conditions that can stabilize public behaviour, thus potentially allowing resistant mutants to thrive. In this paper we theorize on how these different mechanisms can influence the evolution of resistance against public good inhibitors. Based hereon, we propose an innovative 5-step screening strategy to identify novel evolution-proof public good inhibitors, which involves a systematic evaluation of the exploitability of public goods under the most relevant experimental conditions, as well as a careful assessment of the most optimal way to interfere with their action. Overall, this opinion paper is aimed to contribute to long-term solutions to fight bacterial infections.

## Introduction

The discovery and development of antibiotics in the 20th century allowed us to successfully treat most bacterial infections. However, this success also resulted in the overuse and misuse of antibiotics, which strongly accelerated the development and spread of resistance (André and Godelle 2005; Hughes and Karlén 2014; O'Neill 2016). Meanwhile, multiple pathogens turned into multidrug resistant ‘superbugs’, resilient to most or even all our available antibiotics (O'Neill 2016; Mcewen and Collignon 2017). Together with a shortage of long-term investment in the development of new antibiotics, this preludes an alarming lack of effective antibiotics to challenge infections (Hughes and Karlén 2014; O'Neill 2016) and invokes an urgent need for a new generation of antimicrobial drugs (Hughes and Karlén 2014).

The high selection pressure for resistance against traditional antibiotics is inherently related to their activity against functions that are essential for the viability of individual bacteria. Novel types of antimicrobials should therefore preferably be directed towards alternative targets in order to avoid the high probability of treatment failure and low sustainability associated with resistance development (André and Godelle 2005; Allen *et al*. 2014). One strategy currently gaining popularity is the use of anti-virulence drugs that disarm pathogens by targeting their virulence factors (Martinez *et al*. 2019). However, although these virulence phenotypes are often non-essential for bacterial survival or growth in nutrient-rich media *in vitro*, they still provide a fitness benefit, either at the site of infection or in other environments (Allen *et al*. 2014). Otherwise one would expect natural selection to have selected against the expression of these virulence factors, which typically entail a significant energy cost to produce. Anti-virulence drugs in general are therefore expected to still select for resistance, albeit slower than traditional antibiotics as selection might be weaker and only occur under the limited conditions where the virulence phenotypes provide a benefit (André and Godelle 2005; Clatworthy *et al*. 2007; Allen *et al*. 2014). Consistently, resistance against anti-virulence drugs, defined as the recovery of the functional virulence factor after exposure to an anti-virulence drug, has already been observed (Hung *et al*. 2005; Allen *et al*. 2014; Ruer *et al*. 2015; Maura *et al*. 2016).

**Figure 1.**
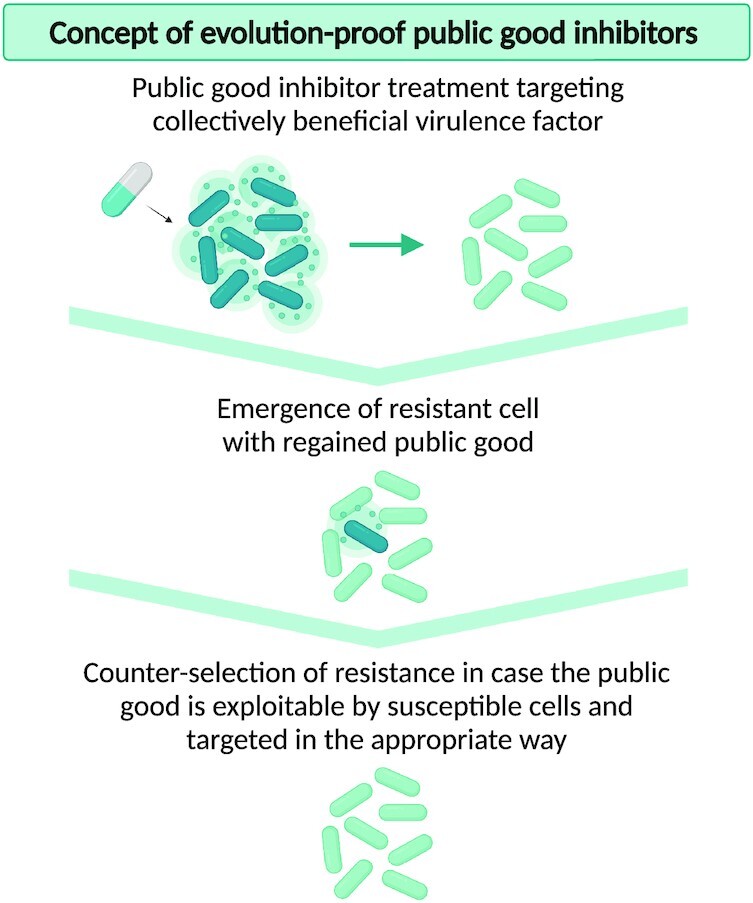
**Schematic illustration of the theoretical concept of evolution-proof public good inhibitors. Top panel:** Public goods are collectively beneficial virulence factors that are costly to produce but provide a shared benefit to other cells in the population. During treatment with a public good inhibitor, the public good will be inhibited in the susceptible population. **Middle panel:** Resistant mutants with regained public good production can emerge in the population. **Bottom panel:** These resistant cells are not expected to be selected for in case the public good is exploitable by susceptible cells, as exploitability implies that (i) resistant cells share the advantages provided by the public good with the surrounding sensitive cells and (ii) susceptible cells experience a net growth advantage compared to the resistant cells because they do not carry the cost of public good production. Such counter-selection of resistance is however only possible if the public good is targeted in the appropriate way, i.e. avoiding public good production in susceptible cells and unwanted side-effects on private traits.

Targeting non-essential virulence traits is thus not sufficient to avoid resistance. However, an ingenious solution has been proposed by social evolution theory. This theoretical framework predicts that targeting the subset of virulence traits that are cooperative in nature can be evolutionarily robust (Allen *et al*. 2014). Such virulence phenotypes make use of what is often known as public goods, shared traits that are costly to produce but benefit other cells in the population. Although resistant mutants can still emerge, these cells are not expected to be selected for as they do not selfishly benefit from their resistance but instead share the advantages provided by the public good with the surrounding sensitive cells (West *et al*. 2006; Allen *et al*. 2014) (Fig. 1). These sensitive cells do not carry the cost of public good production and therefore experience a net growth advantage compared to the resistant cells. Since the sensitive cells thus behave as cheaters that benefit from the public goods produced by the resistant mutants without providing a benefit in return, the sensitive cells outcompete the resistant cooperators (Mellbye and Schuster 2011; Boyle *et al*. 2013; Mitri and Foster 2013). As the frequency of sensitive cells increases, the decreasing amount of cooperative public good produced will no longer suffice to support the whole group ultimately leading to a population collapse, known as Hardin's *tragedy of the commons* (Hardin 1968; West *et al*. 2006).

**Figure 2.**
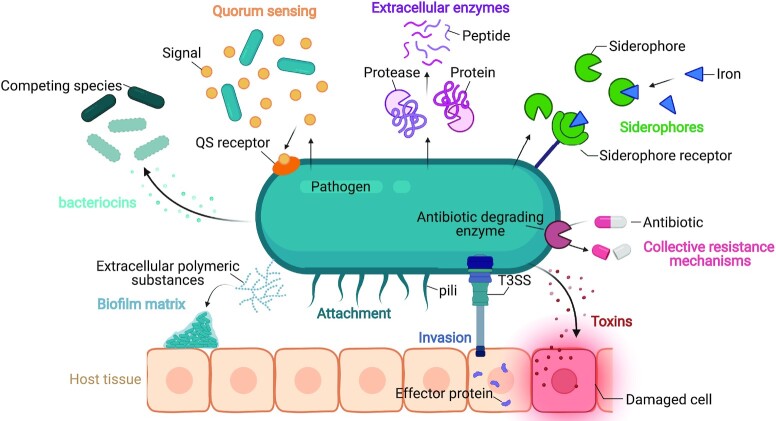
**Illustration of the diversity of publicly beneficial virulence factors present in pathogens and important during infection**. (i) Toxins: molecules damaging host tissue to promote infection and disease play a prominent role in virulence (bottom right). (ii) Invasion: Bacterial secretion systems translocate effector proteins into host tissue, enable bacterial invasion and invoke an inflammatory response (bottom middle). (iii) Adhesion factors and biofilm matrix components: Extracellular proteins (such as pili) and polymers (extracellular polymeric substances) mediate attachment of cells to one another and to host tissue, and provide structure and protection against environmental stressors (bottom left). (iv) Bacteriocins: Antimicrobial molecules produced by microbes decrease the fitness of other bacterial species and aid in competition with host microbiota (left). (v) Quorum sensing signals: Small diffusible signaling molecules mediate bacterial cell-to-cell communication (top left). (vi) Extracellular enzymes: Enzymes mediate digestion of complex molecules (e.g. proteins, polymers) into smaller molecules (e.g. polypeptides, monosaccharides) and aid in nutrient uptake (top middle). (vii) Siderophores: Iron-scavenging molecules, forming soluble Fe^3+^ complexes, allow iron uptake through the cell membrane via specific receptors (top right). (viii) Collective resistance mechanisms: Resistance mechanisms provide public protection and allow the survival of non-resistant bacteria during antimicrobial treatment.

As elaborated in Fig. 2 and Supplementary Table S1, several such publicly beneficial virulence factors have already been described (Nogueira *et al*.^2012^) and are thus potential targets for evolution-proof antimicrobial strategies. Moreover, this concept of interfering with public behaviors is not limited to strategies aimed at disarming pathogens within a host, such as toxins damaging host tissues (O'Loughlin and Robins-Browne 2001; Raymond *et al*. 2012) or the Type III secretion system of *Salmonella* that enables invasion (Diard *et al*. 2013). For instance, also traits that aid in nutrient uptake, e.g. iron-scavenging siderophores (Kramer *et al*. 2020) and extracellular enzymes digesting complex molecules (Greig and Travisano 2004; Diggle *et al*. 2007; Maclean and Brandon 2008; Gore *et al*. 2009; Drescher *et al*. 2014), can be public in nature. Several other public traits enhance tolerance to antimicrobials and the host's immune system (West *et al*. 2007), including structural extracellular polymeric substances of the biofilm matrix (Nadell and Bassler 2011a; Kim *et al*. 2014; Nadell *et al*. 2015; Irie *et al*. 2017; Dieltjens *et al*. 2020), proteins involved in adhesion (Rainey and Rainey 2003; Schluter *et al*. 2015), or resistance mechanisms that break down antibiotics such as β-lactamases (Dugatkin *et al*. 2005; Perlin *et al*. 2009; Medaney *et al*. 2016; Sorg *et al*. 2016; Domingues *et al*. 2017). Even phenotypes involved in competitive interactions have been described as public goods since the removal of a competitor from a niche is beneficial to the complete residual population. Interference with such traits thus not only allows to block virulence, but also to inhibit bacterial growth or survival in an evolutionarily robust manner. Furthermore, this concept also holds true for interference with specific microbial communication systems, such as quorum sensing (QS) (Schuster *et al*. 2013). In this case, the regulated traits do not have to be public themselves, as long as the communication system employs public signals that are costly to produce (Allen *et al*. 2014). In what follows, the term ‘virulence factor’ will therefore refer to all factors that are non-essential for *in vitro* bacterial growth in nutrient-rich media but provide a benefit *in situ*.

Despite this extensive body of work on public goods, only a limited number of pioneering studies, focusing only on a small selection of public goods, have been directed towards providing support for the promising perspective of evolutionarily robust public good inhibitors (Mellbye and Schuster 2011; Ross-gillespie *et al*. 2014; Sully *et al*. 2014; Gerdt and Blackwell 2014a; Rezzoagli *et al*. 2018; Dieltjens *et al*. 2020). A first set of studies focused on the situation where a resistant strain emerges in a treated population and resumes public good production. They mimicked this competition between an emerging resistant strain and surrounding sensitive bacteria by competing a low number of wild type public good producers (mimic of resistant strain) with a higher proportion of deletion mutants unable to produce the public good (mimic of sensitive strain) (Mellbye and Schuster 2011; Gerdt and Blackwell 2014b). Specifically, it was shown that QS-deficient mutants of *P. aeruginosa* can indeed behave as social cheaters and are more fit than QS-cooperators, suggesting that selection against resistance would occur (Mellbye and Schuster 2011; Gerdt and Blackwell 2014b). Although this indicates that an evolution-proof antimicrobial strategy against public goods is theoretically possible, these studies do not account for possible side-effects if public good inhibitors are applied. Others went one step further and used experimental evolution to study resistance in a sensitive bacterial population in the presence of a public good inhibitor (Ross-gillespie *et al*. 2014; Sully *et al*. 2014; Rezzoagli *et al*. 2018). These studies showed that inhibiting QS by savarin in *Staphylococcus aureus* (Sully *et al*. 2014) or pyoverdine siderophores by gallium in *P. aeruginosa* (Ross-gillespie *et al*. 2014) is evolutionarily robust, although it was not directly demonstrated that resistant strains were counter selected. To date, only a single study showed that resistance against a public good inhibitor is counter selected, by combining both aforementioned approaches with a competition experiment between a resistant and sensitive strain in presence of the inhibitor (Dieltjens *et al*. 2020). Here it was shown that the exopolymeric substances (EPS) of *Salmonella* biofilms are a public good that are exploitable by non-producers, that resistance does not evolve under 40 days of EPS inhibitor treatment, and, most importantly, that a resistant strain is outcompeted by a susceptible strain under EPS inhibitor treatment, explaining why resistance does not evolve (Dieltjens *et al*. 2020). Together, these studies provide significant support for the evolutionary robustness of public good inhibitors (Mellbye and Schuster 2011; Ross-gillespie *et al*. 2014; Sully *et al*. 2014; Gerdt and Blackwell 2014b; Dieltjens *et al*. 2020).

In contrast, a number of other studies did observe a spread of mutants resistant against public good inhibitors. However, in each of these cases, the selection for resistance could at least partly be attributed to aspecific effects of the applied public good inhibitor on private functions (Maeda *et al*. 2012; Rezzoagli *et al*. 2018; Imperi *et al*. 2019). Due to this effect on private traits resistance can develop similarly as in the case of classic antimicrobials. Specifically, Maeda *et al*. (2012) showed that resistance to the well-characterized QS-inhibitor furanone C-30, which interferes with the LasR QS-pathway, can spontaneously arise and spread in *P. aeruginosa* populations via enhanced drug efflux (Maeda *et al*. 2012). However, this resistance development can be attributed to the involvement of LasR not only in the regulation of public virulence factors such as exoprotease, biofilm formation, and chitinase (Hentzer *et al*. 2003), but also the regulation of a private degradative enzyme necessary for adenosine-dependent pathogen growth (Heurlier *et al*. 2005), as might be encountered during infection of a host (Maeda *et al*. 2012). Additionally, Rezzoagli *et al*. (2018) probed the evolutionary robustness of flucytosine, targeting the siderophore pyoverdine in *P. aeruginosa*. Long term treatment with flucytosine repeatedly resulted in resistance evolution, probably due to its deleterious off-target effects on RNA synthesis. In accordance with these findings, flucytosine-insensitive *P. aeruginosa* mutants were also selected during co-culturing experiments with the sensitive wild-type strain (Imperi *et al*. 2019).

A much higher number of studies have focused, however, on competition between public good producers and non-producers outside the context of resistance evolution. Most importantly, these studies revealed several mechanisms that can counteract public good exploitation and as such stabilize cooperation (Nadell *et al*. 2016; Steenackers *et al*. 2016; Asfahl and Schuster 2017; Smith and Schuster 2019). Overall, these studies indicated that both the production cost and the shareability of the benefit determine the exploitability of the public good and that stabilizing mechanisms act on both these aspects in a way that is strongly dependent on the environmental conditions (Asfahl and Schuster 2017; Smith and Schuster 2019). Since some of these mechanisms might also influence the exploitation of strains resistant to public good inhibition, they provide a warning that, next to side effects of the public good inhibitor on private traits, additional factors will likely counteract the evolutionary robustness of public good intervention strategies.

Altogether, it is clear that interference with public goods has promising prospects, but that under certain circumstances evolution for resistance might still occur. The goal of this paper is therefore to provide an extensive evaluation of all factors, both mechanistic and ecological, that determine the evolutionary robustness of public good inhibiting strategies. We theorize on how the different public good stabilizing mechanisms known can influence the evolution of resistance against public good inhibitors and analyse how these mechanisms are contingent on the ecological conditions (Section A). We then use the outcome of our analysis to propose a 5-step strategy that allows to identify novel evolution-proof antimicrobial strategies targeting public goods and that takes potential pitfalls already into account in the first stages of screening (Section B). This strategy can be utilized to evaluate the evolution-proof character of strategies targeting known public goods as for several public goods the production cost and shareability have not yet been sufficiently evaluated under relevant conditions (Supplementary Table S1). In addition, we hypothesize that there are also a multitude of unexplored public goods to be mined since their expression is often strongly dependent on the ecological conditions (Kümmerli *et al*. 2009b; de Vargas Roditi *et al*. 2013; Garcia-garcera and Rocha 2020) and these conditions are typically not incorporated in screenings for new antibiotic targets. Moreover, recent advances in secretomics and exoproteomics predict that secreted -and therefore potentially cooperative- proteins account for a significant proportion of microbial proteomes, even up to 40% in some species (Saleh *et al*. 2001; Song *et al*. 2009; Armengaud *et al*. 2012). A substantial percentage of these exoproteomes are hypothetical proteins (González *et al*. 2007; Clair *et al*. 2010; Fernandes *et al*. 2014; Bonar *et al*. 2015; Cheng *et al*. 2017; Sauvage and Hardouin 2020), in addition to yet identified virulence factors (Nogueira *et al*. 2012). Therefore, the strategy we propose is designed to be applicable to both an open screening approach aimed at identifying novel public targets and a targeted screening approach aimed at studying the potential of already known public goods as targets for evolutionarily robust interference strategies. In general, we emphasize that screening methods for novel public good targets of evolution-proof antimicrobial strategies should not only include test systems to examine the benefit of the public good, but should also study the exploitability under relevant conditions.

## A| several factors define the evolutionary robustness of public good inhibitors

As mentioned above, public good cooperation is susceptible to exploitation by non-producing cheaters, which are able to benefit from the public good while lacking the production cost and therefore experience a net fitness advantage compared to producers. In nature, public good cooperation can however be stabilized, because bacteria make use of a multitude of mechanisms to protect against non-producing cheaters, which is the topic of several recent reviews (Asfahl and Schuster 2017; Smith and Schuster 2019). These mechanisms act on both relative benefit and relative cost experienced by producers compared to cheaters and are strongly condition-dependent (Kümmerli *et al*. 2009b; de Vargas Roditi *et al*. 2013). Presumably, the same or very similar mechanisms also control the evolutionary stability of novel or less-characterized public goods as they act on the fundamental aspects of public behavior. However, not all previously identified public good stabilizing mechanisms are expected to be equally relevant for the focal case of resistant strains emerging at low frequency amid sensitive cells under public good inhibitor treatment. Therefore, for each of these known mechanisms, we will motivate their expected impact on the concept of evolutionarily robust drugs (Table 1).

**Table 1.** Mechanisms known to stabilize public good cooperation and their expected consequences for evolutionary robustness of antimicrobial strategies interfering with public good production.

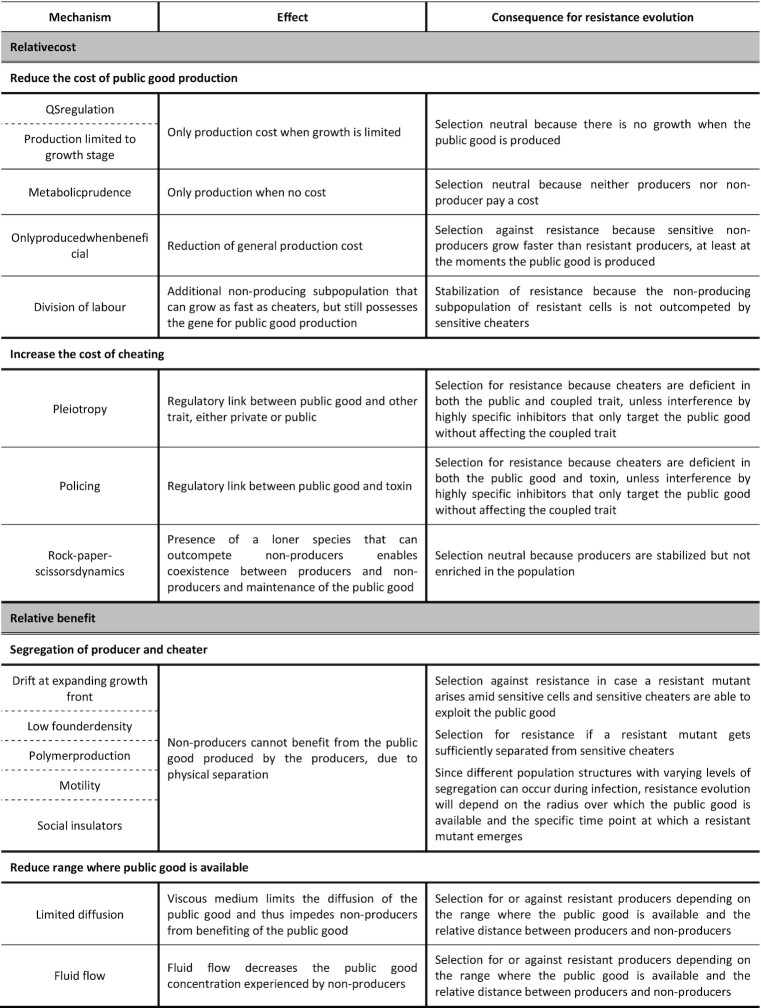
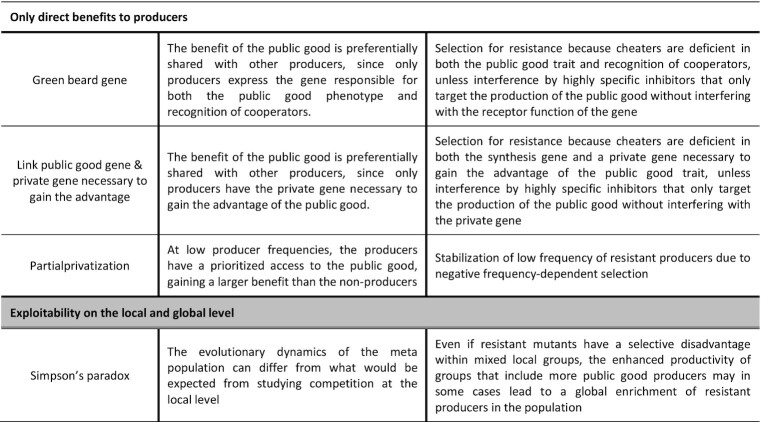

In this section, we will organize the mechanisms and ecological conditions that determine local exploitability of previously characterized public goods based on their influence on relative cost or benefit and discuss their expected impact on resistance evolution. We hereby assume that the public good is sufficiently shared when discussing mechanisms that influence the relative cost and we assume that the cost of public good production is significant when evaluating the mechanisms that determine its relative benefit. In addition, we will motivate that the evolutionary dynamics at the local level cannot just be generalized to the global meta-population, as the Simpson's paradox highlights that both dynamics do not necessarily coincide (Penn *et al*. 2012). In section B we will discuss how the subset of mechanisms relevant for evolution-proof public good inhibitors can be integrated in a screening set-up.

### Relative cost

In order for non-producers to have a fitness advantage over public good producers, the producers need to experience a significant fitness cost. The production cost will differ greatly between the various public goods depending on their inherent properties. However, even if the inherent cost is significant, several mechanisms can reduce the cost of production. In addition, there might also be a cost associated with cheating, which can be further increased by mechanisms that harm non-producers. If a resistant mutant can exert mechanisms to reduce its cost of public good production or cause a disadvantage to non-producers, there will be no selection against resistance. The mechanisms and conditions influencing the fitness cost thus need to be taken into account while evaluating a public good's suitability as target for an evolution-proof antimicrobial strategy.

#### Reduce the cost of public good production

Several of the stabilisation mechanisms for cooperation reduce the cost of producing the public good via regulatory mechanisms that limit the conditions under which the public good is produced. These adaptations of course enhance the fitness of public good producers in general, but they also limit the relative fitness benefit of non-producers and thus stabilize cooperation (Asfahl and Schuster 2017).

Bacteria can regulate public goods by QS, which allows them to restrict public good production to populations with sufficiently high densities. Under these conditions, there is not much potential for growth and thus for cheating to occur (Asfahl and Schuster 2017; Özkaya *et al*. 2017). This density-dependent QS control has been confirmed both *in silico* and *in vitro* with the modified β-lactamase exoenzyme BlaMs in *Escherichia coli* (Pai, Tanouchi and You 2012). Applied to the concept of public good inhibitors, this means that as long as the quorum is not reached, resistant mutants will likely be selection neutral. However, once production of the public good commences, selection against resistance can occur as long as there is still capacity to grow and the public good is sufficiently shared with surrounding sensitive bacteria. This mechanism alone is thus not expected to compromise the evolutionary robustness of strategies interfering with public goods. A similar effect occurs when the public good production is dependent on the growth stage, limiting its susceptibility to exploitation to these specific time points. Ghoul *et al*. for instance showed that pyoverdine production in *P. aeruginosa* - and hence the selective fitness advantage of non-producers - is restricted to the lag phase and early exponential phase (Ghoul *et al*. 2016), whereas competition during the stationary phase is selection neutral. In addition, facultative regulation of public good production can also be based on environmental cues to ensure that the public good is only produced when it provides a benefit. Production of the iron-scavenging siderophore pyoverdine of *P. aeruginosa*, for example, is regulated in response to iron availability, with increased production when iron is limited (Buckling *et al*. 2007; Kümmerli *et al*. 2009b). As a consequence, cheaters only experience a relative fitness advantage in iron-limited environments, but not in iron-rich environments (Kümmerli *et al*. 2009b). Reassuringly, also the latter two mechanisms will not support the spread of resistance as exploitation can still take place once production of the public good commences.

Alternatively, the cost of public good production can be reduced by limiting the production to conditions where the resources necessary to produce the public good are non-limiting. The production of the biosurfactant rhamnolipid, for instance, was found to be suppressed when carbon, the main building block of rhamnolipids, is the limiting nutrient whereas the production was enhanced if nitrogen availability limits growth (Xavier *et al*. 2011). This prudent regulation according to nutrient availability was also reported for the extracellular enzymes elastase and aminopeptidase, the phenazine antibiotic pyocyanin (Mellbye and Schuster 2014), and pyoverdine (Sexton and Schuster 2017). In contrast to the previous mechanisms, the lack of significant growth costs for public good producers holds true even when the public good is produced, rendering inhibition of these public goods selection neutral at best.

Finally, division of labour can also protect against cheaters. In most cases, division of labour can emerge if a part of an isogenic population will not produce the public good due to phenotypic heterogeneity. As this non-producing subpopulation grows as fast as the cheaters, it will not be outcompeted. In contrast to cheaters, this non-producing subpopulation still passes on the gene for public good production to its kin, thus evolutionarily stabilizing the public good (Diard *et al*. 2013). Mutants that acquired resistance to the public good inhibitor and also regained division of labour, can therefore likely not be completely removed from a population by cheating sensitive cells.

Overall, the above mechanisms that reduce the production cost are not deemed to compromise the prospect of evolution-proof drugs. As long as the public good is sufficiently shared, they will only reduce the conditions under which resistance is counter selected and in the worst case render resistance selection neutral entirely. Nevertheless, these mechanisms shouldn't be neglected, since they might pronounce the effect of additional public good stabilizing mechanisms, such as those that reduce public good shareability.

#### Increase the cost of cheating

In addition to lowering the production cost of the public good, bacteria can also stabilize public good production by increasing the cost of cheating. This additional cost imposed on cheaters limits their net relative fitness benefit over producers, thereby preventing exploitation.

One such mechanism is pleiotropy, wherein a single allele is responsible for multiple traits. If the gene responsible for public good production is coupled to private traits, cheaters will lack access to benefits provided by these private traits as these are not shared by the producers (Mitri and Foster 2013; Asfahl and Schuster 2017). For instance, a pleiotropic linkage via the *dimA* gene during fruiting body formation in the social amoeba *Dictyostelium discoideum* ensures that defectors who do not contribute to the supporting stalk will be excluded from the reproductive spores (Foster *et al*. 2004). Similarly, co-regulation of genes encoding for public and private traits through QS constrains exploitation of the QS-controlled public good under specific growth conditions (Wilder *et al*. 2011; Dandekar *et al*. 2012). In this context, the brominated furanone C-30 inhibits QS in *P. aeruginosa* and hereby represses many public QS-controlled virulence factors such as protease, pyoverdine and chitinase (Hentzer *et al*. 2003). However, it also inhibits a private degradative enzyme, nucleoside hydrolase, which is necessary for growth in minimal medium with adenosine as the sole carbon source (Heurlier *et al*. 2005). When grown in such a medium, mutants that acquire resistance to C-30 by restoration of LasR pathway activity, not only regain access to the public traits but also the private benefit and were, consistently, found to experience a positive selection pressure (Maeda *et al*. 2012). Besides, a pleiotropic linkage between two different public goods might in some settings also keep cheaters in check and shape the evolutionary dynamics of each public good trait. For example, siderophore production in *P. aeruginosa* is fine-tuned depending on the relative iron availability (Ross-Gillespie *et al*. 2015). Under strong iron limitation, bacteria invest in the metabolically more costly but more effective iron chelator pyoverdine, while the presence of pyoverdine directly suppresses production of the less effective but cheaper siderophore pyochelin. However, a strong negative feedback causes a shutdown in pyoverdine synthesis when iron is more readily available, resulting in a switch to pyochelin production under moderate iron limitation (Dumas *et al*. 2013; Ross-Gillespie *et al*. 2015). This negative regulatory linkage between both siderophores under strong iron limitation, however, also ensures that pyoverdine-negative cheats become pyochelin-producing cooperators, diminishing their relative fitness benefit over pyoverdine producers and therefore counteracting exploitation of pyoverdine (Ross-Gillespie *et al*. 2015).

Public goods may also be co-regulated with toxin-immunity systems, which is referred to as policing. This joint regulation allows cooperators to directly harm cheaters that do not produce the public good, since cheaters will be sensitive to the toxin secreted by cooperators (Asfahl and Schuster 2017; Smith and Schuster 2019). To illustrate, in *P. aeruginosa* the LasR-LasI QS-system controls the exoprotease elastase as well as the RhlR-RhlI QS-system, which in turn controls the production of and immunity to antimicrobials such as phenazines and cyanide (Smalley *et al*. 2015; Wang *et al*. 2015). Consequently, regulation-deficient cheaters suffer a fitness cost due to their sensitivity to cyanide produced by the cooperators (Wang *et al*. 2015). If the public good is a toxin in itself, it suffices that toxin production is linked to the immunity gene. For example, *Burkholderia thailandensis* can inhibit cheaters that lack the contact dependent growth inhibition systems (Anderson *et al*. 2014).

In general, such mechanisms that couple the production of a public good to other traits will prevent exploitation by cheaters in case the cheaters are deficient in the whole regulatory pathway, including both the public and the coupled trait. However, cheaters that are deficient downstream in the pathway and are only affected in the public good and not in the coupled trait, will still be able to exploit producers. The evolutionary robustness of inhibitors of coupled public goods will therefore strongly depend on the position of their target within the pathway. As discussed in detail in section B, highly specific inhibitors that only target the public good without affecting the coupled trait are the preferred option. In this case, sensitive strains still express the coupled trait, as such ensuring that exploitation of the resistant producer can occur. On the contrary, if the inhibitor acts on an upstream regulator of both public and coupled trait, selection for resistance will occur if both the coupled private trait and public good are restored in the resistant mutant.

Alternatively, cheaters can also experience an extra cost compared to producers due to the presence of a third - so-called ‘loner’ or ‘modulator’ – strain. If in such a community the cooperators outcompete the loners, the loners outcompete the cheaters and finally the cheaters outcompete the cooperators, this will result in cyclical rock-paper-scissors dynamics (Kerr *et al*. 2002; Kelsic *et al*. 2015; Inglis *et al*. 2016). The presence of these loner species thus enables coexistence between cheaters and cooperators and maintenance of the public good production in the population. Such rock-paper-scissors dynamics have been demonstrated for bacteriocin-producing (cooperator), -resistant (cheater) and -sensitive (loner) strains in a structured *E. coli* community (Kerr *et al*. 2002) as well as in simulations with a public antibiotic-degrading resistance mechanism in well-mixed populations (Kelsic *et al*. 2015). Moreover, Inglis *et al*. observed rock-paper-scissor dynamics among pyoverdine producers, cheaters and a loner strain which produces its own siderophore for iron-uptake in both simulations and evolution experiments of well-mixed *P. aeruginosa* communities (Inglis *et al*. 2016). Interfering with public goods in a rock-paper-scissors community should thus not result in extinction of resistant mutants but rather in oscillatory dynamics between the resistant, sensitive and loner strains.

### Relative benefit

Besides lowering the relative cost of public good production, public good production can also be stabilized by reducing the relative benefit for non-producers (Asfahl and Schuster 2017). If cheaters benefit to a lesser extent from the public good than the producers, they may not be able to outcompete the producers and inhibition of the public good might not lead to an evolution-proof antimicrobial strategy (West *et al*. 2006). This limited access of cheaters to the public good might be achieved by (i) physically separating producers from non-producers, (ii) reducing the distance over which a secreted public good provides its benefit or (iii) selectively directing the benefit to other producers.

#### Segregation of producer and cheater

In order for non-producers to benefit from public goods, they must be in sufficiently close proximity of the producers and the collective benefit they provide. A first factor determining the distance between producers and non-producers is population density. Overall, low densities result in weaker interactions and less access to the public good for non-producers (Darch *et al*. 2012). In structured environments such as biofilms, spatial segregation between producers and non-producers is also a crucial factor that influences proximity. Translated to our concept of evolution-proof public good inhibitors, this implies that if a resistant mutant gets sufficiently separated from sensitive cheaters, this resistant cell is expected to thrive and give rise to a resistant population, as has been shown generically for emerging cooperating cells by in *silico* models (Melbinger *et al*. 2015). On the other hand, if the resistant cell arises amid sensitive cells, the public goods produced by this mutant likely can readily be exploited by sensitive bacteria. Next to these two extreme cases of complete segregation versus complete mixing, populations with varying levels of segregation can arise and affect exploitation of public goods differently depending on the radius over which the public good is available. Next to influencing the interaction between producers and non-producers directly, the level of spatial structuring was also found to affect the abovementioned rock-paper-scissors dynamics with third ‘loner’ strains (Kerr *et al*. 2002). Known factors determining the level of genotypic segregation and subsequent stabilization of cooperative phenotypes in structured environments are drift in expanding growth fronts, founder density, and polymer secretions. Most of these mechanisms have recently been reviewed by Steenackers *et al*., Nadell, Drescher and Foster and Yanni *et al*. (Nadell *et al*. 2016; Steenackers *et al*. 2016; Yanni *et al*. 2019) and will only be briefly discussed here.

First, low nutrient availability, low substrate diffusion, or fast growing cells can thin the layer of active cells at the biofilm border due to limited nutrient availability in the deeper regions (Nadell *et al*. 2010; Mitri *et al*. 2016; Frost *et al*. 2018). These limited numbers of active cells are subsequently subjected to random genetic drift, resulting in sectoring and segregation (Nadell *et al*. 2010; Steenackers *et al*. 2016). Second, Van Gestel *et al*. showed that a low founder cell density enhances segregation within structured populations of *Bacillus subtilis* as cells are initially more separated and more likely proliferate into separate subpopulations each containing a single genotypic lineage (Van Gestel *et al*. 2014). Such segregation induced by low founder density also impeded pyoverdine exploitation by cheaters in *P. aeruginosa* (Ross-gillespie *et al*. 2009) and lowered the fitness of β-lactam sensitive *E. coli* cells compared to resistant β-lactamase producers when co-cultured under ampicillin treatment (Domingues *et al*. 2017). Polymer production can also influence population structuring by the formation of tower-like structures dominated by certain lineages, as such physically separating different lineages from each other (Xavier and Foster 2007; Nadell and Bassler 2011b; Kim *et al*. 2014). Moreover, certain polymers, such as RbmA in *V. cholerae*, enhance adhesion between mother and daughter cells. This mother-daughter adhesion enhances the interaction between relatives and segregates different cell lineages, hereby increasing the distance between cooperators and cheaters. Subsequently, cheaters are displaced and excluded from the biofilm (Nadell *et al*. 2015). A similar mechanism of differential adhesive strengths has been suggested as an explanation for the observation that production of the *P. aeruginosa* polysaccharide PSL by PSL+ strains could not successfully be exploited by PSL- cheaters (Irie *et al*. 2017). Also motility can impact population structure (Mitri *et al*. 2011) in various manners, based on its complex interactions with cell adhesion (Martínez-garcía *et al*. 2018), population density (Martínez-garcía *et al*. 2018) and the environmental fluid flow (Martínez-garcía *et al*. 2018; Rossy *et al*. 2019). Finally, the presence of other species can influence the segregation between producers and non-producers of the focal species (Mitri *et al*. 2011; Steenackers *et al*. 2016). For example, other species can act as social insulators and form a spatiogenetic barrier between the cooperators and the cheaters, limiting the access of cheaters to the public good (Mitri *et al*. 2011). However, this insulation effect requires limited niche overlap between the two species, otherwise the other species could act similarly to cheaters, further destabilizing public good production. In addition to the social insulation, other species could also influence the spatiogenetic structure of a microbial community via effects of competition. For instance, Type VI secretion system-mediated killing in *Vibrio cholerae* has been shown to induce segregation and favour the evolution of public good cooperation (Mcnally *et al*. 2017).

#### Reduce range where public good is available

Environmental conditions can also prevent non-producers of accessing the public good by limiting the distance over which a secreted public good provides its benefit. Hence, the benefit will be confined to the producer and its nearby clone mates, restricting exploitation (Drescher *et al*. 2014). Viscous environments for instance, can limit the diffusion and impede non-producers from benefiting of the public good (Kümmerli *et al*. 2009a; Drescher *et al*. 2014), as is the case for the extracellular enzyme chitinase that degrades the solid polymer chitin into soluble GlcNAc oligomers. If chitinase producers are located in thick, densely-packed biofilms, the GlcNAc oligomers will be exhausted by neighbouring producer cells before reaching the non-producers (Drescher *et al*. 2014). Similarly, the diffusion of the pyoverdine and pyochelin siderophores in *P. aeruginosa* decreases with increasing viscosity of the growth medium, enhancing the relative fitness benefits of siderophore producers in viscous medium (Kümmerli *et al*. 2009a).

In addition to viscosity, fluid flow can also decrease the public good concentration experienced by non-producers. Even at low flow rates, the GlcNAc oligomers produced by chitinase producers were found to be transported away from the surface of the *V. cholerae* biofilm, restraining access to non-producers (Drescher *et al*. 2014). However, the effect of flow is public good-specific since in the case of QS-controlled biofilm growth (Popat *et al*. 2012) and resistance mechanisms (Rojo-Molinero *et al*. 2019), non-producers were found to still show a higher relative fitness than producers in conditions with flow. These results further demonstrate that one cannot generalize the results of one specific public good to other public goods and that the condition-dependency must be determined for each case individually.

#### Only direct benefits to producers

Finally, certain microbes are able to selectively direct the benefit of their public good production to other producers, irrespective of the environmental conditions or composition of the population. Certain genes expressing a public good have been shown to also actively mediate recognition of other public good producing cells and direct the public benefits towards these cells (Jansen and Van Baalen 2006). Such so-called green beard genes have been experimentally found in *Saccharomyces* yeast strains when studying their cooperative biofilm-like behaviour termed flocculation. It was shown that *Saccharomyces cerevisiae* cells expressing the FLO1 gene co-flocculate with other FLO1-expressing *Saccharomyces* cells while excluding non-expressing *flo1* cells (Smukalla *et al*. 2008; Belpaire *et al*. 2021). It is unlikely that public goods encoded by green beard genes will provide suitable targets for evolutionarily robust antimicrobial strategies. Indeed, this would require the identification of inhibitors that only inhibit the production of the public good without interfering with the recognition function of the gene in order for the public good to be exploitable by non-producers.

A similar mechanism to direct public goods to the producers can be found in a genetic linkage between the synthesis gene for the cooperative trait and a private gene which is necessary to gain advantage of the same cooperative trait. For example, co-expression of the synthesis gene and the specific receptor gene of the siderophore ornibactin on the same operon stabilizes cooperation in *Burkholderia cenocepacia* (Sathe *et al*. 2019). In order for an antimicrobial strategy to select against resistance, interfering with such a public good requires specific inhibition of the synthesis function without inhibiting the receptor function, as this implies that sensitive strains can still benefit if a resistant cell regains the synthesis function. If the inhibitor targets both functions at the same time, for example via an upstream regulator, a resistant strain should regain both traits to become resistant. However, since the cells sensitive to such an inhibitor do not possess the receptor function, they will not be able to exploit the public good reproduced by a resistant cell and selection for resistance will occur.

Partial privatization can also increase the relative benefit for producers as the producing cells will have a prioritized access to the public good. In cases where these privatization mechanisms are strong, cheaters might not be able to outcompete the producers, as was described for the PSL polysaccharide in *P. aeruginosa* biofilms (Irie *et al*. 2017). In other cases partial privatization might result in a negative frequency dependent selection of public good producers. At low producer frequency, the concentration of the public good is low, which is expected to amplify the effect of mechanisms that privatize the benefits to producers. In contrast, at high producer frequency, the concentration of the public good might be sufficiently high to saturate the beneficial effects and allow non-producers to benefit at (near) maximum efficiency even though privatization mechanisms are in place. This more pronounced effect of privatization at lower producer frequencies then leads to a negative frequency-dependent selection of producers, a phenomenon that has been widely demonstrated for various public goods (Ross-gillespie *et al*. 2007; Gore *et al*. 2009; Rumbaugh *et al*. 2009; Wilder *et al*. 2011; Raymond *et al*. 2012; Yurtsev *et al*. 2013; Scholz and Greenberg 2015; Domingues *et al*. 2017; Aijaz and Koudelka 2019; Amanatidou *et al*. 2019). Examples are the extracellular enzyme invertase (Gore *et al*. 2009), the TEM-1 β-lactamase enzyme (Yurtsev *et al*. 2013) and the siderophore enterochelin (Scholz and Greenberg 2015). When interfering with a public good that is partially privatized at low producer frequencies, selection for resistance will thus most strongly occur when resistant cells are rare and sensitive cells might not be able to completely outcompete the resistant cells (Perlin *et al*. 2009; Yurtsev *et al*. 2013; Sexton and Schuster 2017). This can result in a stable coexistence between producers and non-producers at low producer frequencies (Ellis *et al*. 2007; Gore *et al*. 2009; Raymond *et al*. 2012; Yurtsev *et al*. 2013; Allen *et al*. 2016). Whether stabilization of a low frequency of resistant producers in the population will cause treatment failure, depends on whether the residual amount of public good retained through privatization will be sufficient to allow bacterial virulence. In the event that partial privatization mechanisms at low frequencies would suffice to maintain disease, a change in conditions might still result in a successful treatment. If biofilm EPS are targeted for instance, a population consisting of only a few producers will be inadequate to produce a fully protective biofilm and might still be sensitive to clearance by the immune system and antimicrobial treatment (Dieltjens *et al*. 2020). However, note that if one of these remaining resistant mutants succeeds to get isolated, it will thrive as no cheaters are present, and selection for resistance will occur.

### Exploitability on the local and global level: simpson's paradox

It is important to note that in some cases the evolutionary dynamics of the meta-population might differ from what would be expected from studying competition at the local level. This meta-population analysis is especially valuable for populations with a high degree of dispersal, leading to several subpopulations with different relative compositions of resistant and sensitive strains (Penn *et al*. 2012; Melbinger *et al*.^2015^). Even if cooperators and thus mutants resistant to the therapy have a selective disadvantage within these mixed local groups, the enhanced productivity of groups that include more public good producers may in some cases lead to a global enrichment of resistant producers in the meta-population (Penn *et al*. 2012; Melbinger *et al*. 2015; Penn 2015). This phenomenon is known as Simpson's paradox and has been demonstrated *in vitro* in artificially constructed groups of *E. coli* Rhl producers and non-producers at low founder densities (Chuang *et al*. 2009). However, no evidence of Simpson's paradox was found in *in situ* microcolonies of *P. aeruginosa* siderophore producers and cheats (Penn *et al*. 2012). It is therefore hard to predict to which extent Simpson's paradox will be relevant in the context of specific treatments. This paradox however stresses the importance of studying the competition both at the global population level and in local patches.

## B| an innovative screening method to identify evolution-proof inhibitors of public good cooperation

**Figure 3.**
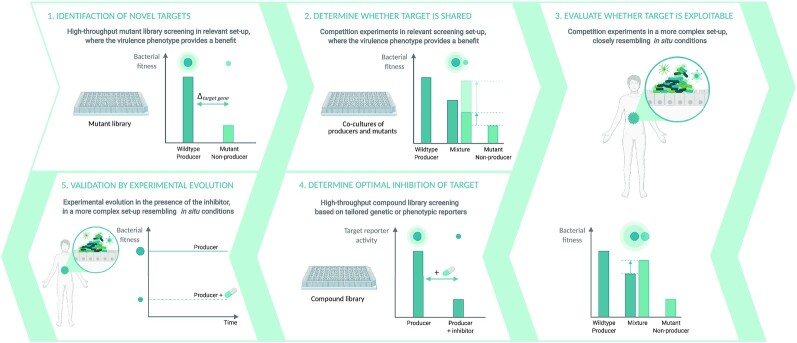
Overview of the proposed 5-step screening approach to identify novel evolution-proof antimicrobial strategies targeting public goods. Step 1 involves a high-throughput screening of a mutant library in simplified, but relevant conditions to identify novel targets for anti-virulence strategies. Step 2 determines the public character of the identified virulence factors by evaluating whether non-producers are able to benefit from the presence of producers. The potential for evolutionary robustness is then investigated in step 3 by evaluating the exploitability of the public good in a more complex set-up closely resembling *in situ* conditions. The public good is exploitable if it is shared to such an extent that non-producers outcompete producers in co-culture. Once a promising public good has been identified, the best approach to target the public good in an evolution-proof manner is identified in step 4. Finally, in step 5 the robustness against resistance evolution of the selected inhibitor should be validated in a long term evolution experiment. Afterwards, the identified inhibitors should be validated *in vivo* in more complex animal models and *in situ* in patients during clinical trials, with particular attention to resistance evolution. The size of the dots above the bars indicates the fitness of the respective strain. A glow around the dot indicates that the public good is produced and its size indicates to which extent the public good is shared.

After evaluating the expected impact of public good stabilization mechanisms on the evolutionary robustness of public good interfering strategies, we will now consider how these potential pitfalls for evolution-proof public good inhibitors can be incorporated in a 5-step screening set-up for the identification of novel evolution-proof antimicrobial strategies targeting public goods (Fig. 3). The first step of our *in vitro* screening set-up is specifically directed to the open screening approach to identify novel target public goods; the next 4 steps are shared with the targeted approach focused on specific yet known public goods. This first step involves a high-throughput screening of mutant libraries in relevant conditions to identify novel targets for anti-virulence strategies. The second step evaluates whether non-producers are able to benefit from the production of the identified virulence factors in order to determine the public character of these virulence factors. The potential for evolutionary robustness is then investigated in step 3 by evaluating the exploitability of the public good in relevant conditions. The public good is exploitable if it is shared to such an extent that non-producers not only perform better in the presence of producers than in monoculture (step 2), but that they even outcompete producers in co-culture. Once a promising public good has been identified, the best approach to target the public good production in an evolution-proof manner is identified in step 4. Finally, in step 5 the robustness against resistance evolution of the selected inhibitor should be validated via competition experiments between strains differing in their sensitivity to the inhibitor and long-term evolution experiments. Afterwards, the identified inhibitors should follow the traditional preclinical and clinical stages of drug discovery. Hence, they should be validated *in vivo* in animal models and *in situ* in patients via clinical trials, albeit still with particular attention to resistance evolution.

### STEP 1: Identifaction of novel targets for anti-virulence strategies

The first step of our proposed screening strategy involves the identification of novel targets for anti-virulence strategies. If a suitable virulence factor is already characterized, this first screening step is not required. Prior to a first *in vitro* screening step, computational approaches can aid to predict the number and types of virulence factors present in a specific pathogen based on genomic data (Pincus *et al*. 2020; Rentzsch *et al*. 2020; de Nies *et al*. 2021). Novel targets can then be identified by performing a high-throughput screening of mutant libraries for lower expression of the virulence phenotype and/or reduced fitness under relevant conditions that mimic the infection under focus. The most suited way to interfere with the function of the identified targets is then evaluated in a later stage (step 4). The main advantage of this approach is that it directly yields a combination of isogenic strains (mutant vs. wildtype) that only differ in the production of the public target. These strains can be used to investigate the exploitability of the public good (and thus evolutionary robustness of inhibitors) by performing head-to-head competitions (steps 2 and 3). An alternative approach would be to directly screen a compound library for inhibitors that interfere with virulence factors. In this case, evaluation of the evolutionary robustness relies on acquiring strains resistant against the identified inhibitors (as elaborated in step 5). However, traditional evolutionary strategies often used to create resistant strains cannot be utilized as resistant mutants will not be selected for in case the strategy is successful. In addition, strategies based on compound library screenings yield less information about the exploitability of the targeted virulence factor since resistance evolution will strongly depend on the interference strategy (as elaborated in step 4) and might even be solely attributable to unknown side effects of the inhibitor. Therefore, we opted to focus our ideal strategy around mutant library screening.

We propose two main approaches to screen for knockout mutants causing reduced virulence. A first possibility is to directly measure the effect of the perturbations on the virulence trait. However, since this requires either a standardized read-out of the virulence phenotype or knowledge on the genes involved in the specific phenotype, this method might be difficult to apply in an open screening approach for novel targets. Besides, virulence phenotypes are often challenging to measure in a high throughput manner. A second option is to measure the strain's fitness in relevant conditions where the virulence phenotypes provide a benefit. Although this requires a rather complex set-up that assesses fitness in conditions relevant for the infection, this method provides an easier read-out as it depends on growth or yield measurements. Depending on the complexity of the infection under focus, it might be that such a high-throughput set-up is only feasible when focussing on a specific stage of the infection. In the case of a *Salmonella* gut infection for instance, one could either focus on (i) gut colonization and survival in the gut lumen or on (ii) invasion and survival inside epithelial cells (Wotzka *et al*. 2017). This second method also ensures that the relevant public traits are expressed, as highlighted by a recent study showing that the occurrence of several extracellular proteins is condition-dependent (Garcia-garcera and Rocha 2020). Moreover, if a parallel screening is performed in a simplified virulence-independent set-up in nutrient-rich laboratory conditions, this approach also allows to simultaneously exclude factors that directly affect growth, irrespective of the environment. This higher throughput set-up can eventually be extended in a later stage of the screening where the evolutionary robustness is evaluated and the *in situ* conditions should be mimicked even more accurately (step 3). For purposes of feasibility and control, the proposed screening steps will likely rely on *in vitro* models or strongly simplified *in vivo* models.

### STEP 2: Determine whether the virulence factor is public

The second step aims to evaluate whether the identified virulence factor is public by studying the extent to which non-producers are able to benefit from the public good in the presence of producers. The public character of the virulence factor can easily be evaluated by setting up a co-culture of a wildtype producing strain and the isogenic knockout mutant strains identified in step 1. As mentioned, these strains respectively mimic a resistant strain that can still produce the public good in the presence of a public good inhibitor and a susceptible strain unable to produce the public good under treatment. If the fitness of the mutant strain in this co-culture (step 2) is higher than in monoculture (step 1), this indicates that the targeted virulence factor is public. As we expect the public character of the virulence factor to be less dependent on the conditions than its exploitability (step 3), this step can be performed in the high-throughput screening set-up and is therefore meant to reduce the number of hits before continuing to a more complex system. However, when following a targeted approach with a previously characterized virulence factor or if the first screening step only revealed a relatively low number of hits, this second step can be omitted and exploitability experiments in complex systems (step 3) can be performed directly.

### STEP 3: Evaluate exploitability in relevant conditions

**Figure 4.**
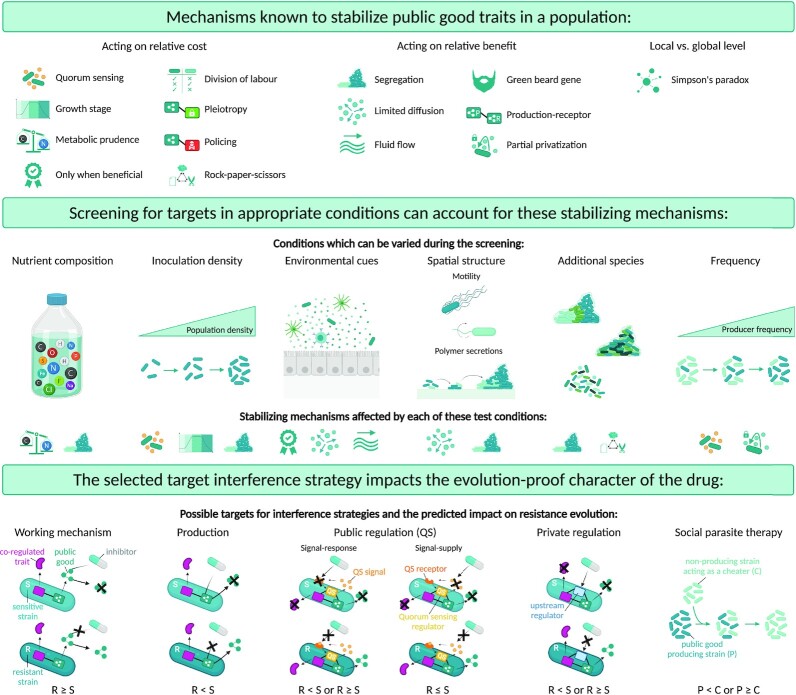
Direct identification of novel evolution-proof treatments implies both appropriate screening conditions that closely mimic the *in situ* situation and an appropriate interference strategy to be able to account for the majority of the previously identified public good stabilizing mechanisms. **Top panel:** Schematic representation of previously identified public good stabilizing mechanisms. **Middle panel:** The potential for evolutionary robustness of strategies interfering with a certain public good target should be investigated in complex set-ups closely resembling the *in situ* conditions. By evaluating the exploitability of the public good under relevant nutrient compositions, inoculation densities, environmental cues, spatiogenetic structures (as attained by varying motility and/or polymer secretions), species compositions and producer frequencies, the relevance of specific public good stabilizing mechanisms can be assessed (as displayed with the corresponding icon of the stabilization mechanism at the bottom of the panel). **Bottom panel:** The specific target of the public good inhibitor impacts resistance evolution, as illustrated by showing the effect of the selected interference strategy on public good production in a cell sensitive to the inhibitor (S) vs. a resistant mutant (R). Moreover, to illustrate whether the selected interference strategy is impacted by stabilization mechanisms that couple the public good to another trait, the effect on production of a possible co-regulated trait is included as well. Although interference with the working mechanism of the public good only affects the public good trait without affecting co-regulated traits, this interference strategy is expected to be selection neutral or to even select for resistant mutants (R ≥ S) since producers will not bear a significantly higher production cost compared to sensitive cells. Interference with the synthesis gene or production mechanism of the public good will counteract stabilization mechanisms that couple the public good gene to another gene, while resistant mutants still encounter a significant production cost compared to sensitive strains, and will thus select against resistance if the public good is sufficiently shared. Resistance evolution following interference with public regulation mechanisms such as quorum sensing (QS) will be different for signal-response versus signal-supply inhibitors. Resistance evolution of the former will depend on the potential co-regulated (private) traits. Signal-supply inhibitors on the other hand are expected to be selection neutral or select against resistance, independent of the regulated trait, since the QS signalling molecules are public themselves and the public good is not produced below the quorum threshold. Resistance evolution upon interference with private regulation mechanisms of the public good will depend on whether the upstream regulator co-regulates other (private) traits. If the inhibitor solely targets the public good without targeting the coupled trait, the selection pressure for resistance will be lower than if both traits are affected simultaneously, as in the case of interference with an upstream regulator. Social parasite therapy, introducing non-producers in the population instead of treatment with a chemical inhibitor, is predicted to select against resistance, but the slow clearance of producers over time nevertheless entails the risk of altered competition patterns driving competition in favour of resistant mutants.

In order for new antimicrobial strategies to be evolutionarily robust, non-producers need to exploit the public good producers and subsequently outcompete them in co-culture. This exploitability can be evaluated by competing wildtype public good producers and an isogenic knockout mutant unable to produce the public good under infection relevant conditions. For a public good to be exploitable, it is not only required that the non-producing mutant performs better than in monoculture, it should also have a higher fitness than the producing wildtype strain in co-culture. As these producing wildtype and non-producing mutant strains are isogenic except for the target gene related to the public good, the outcome of competition is determined by the net relative benefit of the public good, based on both the relative benefit and relative cost of production. Indeed, in order for exploitation to take place, the public good needs to be sufficiently shared with non-producers, allowing them to benefit from the public good at a level comparable to the producers. In addition, even if the public good is shared, the cost of public good production needs to be sufficiently high in order for non-producers to have a significant fitness advantage (West *et al*. 2006). In what follows, we will discuss how the previously reviewed mechanisms and conditions stabilizing public good production (see section A, Table 1) can be implemented in our screening set-up for evolution-proof drugs (Fig. 4).

#### Implementing mechanisms and conditions that influence the relative fitness cost

Since mechanisms that reduce the production cost of public goods might pronounce the effect of other public good stabilizing mechanisms, it is advisable to implement them in the screening set-up. To account for metabolic prudence, the growth medium should have a similar nutrient composition as *in situ* in order to ensure that the same resources are limiting as in the envisioned application. Furthermore, the population density should be in the same range in order to take into account QS or growth stage dependent regulation. Finally, environmental cues that regulate public good production, including host-derived factors and bacterial secretions, should be included in the set-up as well.

Division of labour and the coupling of private and public traits can strongly influence the evolutionary robustness of inhibitors, but do not need to be taken into account separately during step 3 as these mechanisms are inherently incorporated within the genetic circuitries of the microbes under focus. As other species that occupy the same niche can add another level of complexity to the relative fitness cost of public good production, we argue that these additional species should also be included when screening for public goods. Since it is unfeasible, however, to include all possible competitors a focal species might encounter, there will always be a risk for interspecies competition to influence the *in situ* competition between public good producers and non-producers beyond the observations in the lab. This urges the need for further *in vivo* and *in situ* validation after the proposed screening protocol (see step 5).

The combined effect of the cost of public good production and the cost of cheating determines the relative fitness cost for producers compared to non-producers. In order to quantify this relative cost experimentally, the growth rate or yield of producers and non-producers can be compared in conditions where the public good is expressed, but does not yield any advantage (Velicer and Yu 2003; Griffin *et al*. 2004; Diggle *et al*. 2007; Maclean *et al*. 2010; Drescher *et al*. 2014; Srinandan *et al*. 2015; Dieltjens *et al*. 2020). These assays should be performed both in monoculture and mixed culture conditions, as in some cases the cost for cheating might only become visible when producers and cheaters are mixed. For instance, policing mechanisms based on a toxin-antitoxin co-regulation with the public good require mixed culture conditions to determine the cost of cheating. Besides, multispecies conditions could be useful to detect whether the presence of additional species alters the relative fitness cost for producers compared to non-producers. Also computational modelling can further contribute to understanding the influence of the discussed mechanisms by varying the cost of the public good and changing the conditions the microbes are exposed to (Gore *et al*. 2009; Maclean *et al*. 2010; Sexton and Schuster 2017).

#### Implementing mechanisms and conditions that influence the relative benefit

Because population structure is such an important factor influencing public good exploitation, it is critical to incorporate and mimic the spatial structure of the envisioned application when screening for new targets for evolution-proof strategies. Fluorescent *in situ* hybridization on tissue samples could be used to determine the biofilm architecture, distribution of species and spatial parameters at the site of infection (Zijnge *et al*. 2010). However, since it is nearly impossible to unravel the dynamic interplay between all factors contributing to the population structure *in situ*, we propose a strategy where the population structure is observed *in situ* and artificially mimicked *in vitro* by varying known and controllable factors influencing this structure. Founder density, polymer secretion, motility, and presence of additional species can be relatively easily controlled for this purpose, and are preferred above nutrient availability, since manipulating the latter is expected to not only influence spatial segregation but also the production cost and regulation of the public good. Founder density can be varied by changing the inoculation density. Polymer secretion can be adjusted via RNA interference mechanisms (Tomari and Zamore 2005), antisense RNA constructs (Yang *et al*. 2011; Saberi *et al*. 2016) or chemical inhibition (Dieltjens *et al*. 2020), or completely inhibited by constructing knock-out mutants. Similar strategies can be used to modify bacterial motility. Alternatively, chemoattractants can be applied to guide the movement of bacteria (Jain *et al*. 2016). Since different population structures can occur throughout the course of infection, the specific time point at which a resistant mutant emerges could determine whether or not it is selected. It is therefore important to evaluate exploitability of the public good at different possible degrees of spatial segregation. If non-producers have a fitness benefit at the majority of these structures, the risk of resistant mutants to stabilize in the population by thriving locally and creating sufficient segregation with sensitive cells, is strongly reduced.

To account for environmental conditions limiting the distance over which a public good is accessible and exploitation can take place, it is advisable to implement a similar biofilm composition, viscosity and flow rate as *in situ*.

The coupling of public good production to active discrimination mechanisms does not need to be taken into account separately during the screening for exploitable targets as the coupling inherently occurs within the genetic circuitries of the microbes. To test whether frequency-dependent partial privatization mechanisms are in place, we consider it useful to test whether the targeted public good is still exploitable at very low frequencies of producers.

#### Implementing Simpson's paradox

It is highly challenging to integrate meta-population selection effects in an *in vitro* or simplified *in vivo* set-up. However, results obtained in step 3 of the experimental screening set-up could indicate to what extent the Simpson's paradox might influence the selection of resistant mutants at the meta-population level. Simpson's paradox likely applies if (i) the presence of cheaters in the co-culture community strongly diminishes the absolute population fitness as compared to the overall fitness of a producer monoculture and (ii) cheaters only have a small relative fitness benefit over producers in the co-culture (Fig 3, step 3) (Penn *et al*. 2012). If Simpson's paradox is expected, we suggest to explore its potential impact *in silico* and to validate these predictions *a posteriori* via *in vivo* and *in situ* observations of the infection in different host tissues.

### STEP 4: How to inhibit the public good

Once a promising virulence or tolerance associated public good has been identified, the most optimal strategy for inhibition needs to be determined. There are three main approaches drugs can target a public good: (i) by targeting the working mechanism of the public good, (ii) by targeting the production of the public good and (iii) by targeting the public good's regulatory system. Depending on the selected approach, a suited high-throughput compound library screening set-up can be designed based on tailored genetic or phenotypic reporters. This compound library can either be a small molecule library (Hung *et al*. 2005) or a repurposing library with drugs which are already approved for other purposes (Imperi *et al*. 2013; D'Angelo *et al*. 2018). Whether the identified drug provides an evolution-proof strategy is expected to depend on the way it interferes with the public good (Fig. 4).

(i) When interfering with the working mechanism of the public good, both resistant and sensitive cells produce the public good, although only the public good produced by the resistant cells will be effective. Because both the sensitive and resistant strain carry the same production cost, this strategy is not expected to result in a fitness advantage for the sensitive strain. In the best-case scenario, i.e. when the resistant and sensitive strain have equal access to the public good, this strategy will be selection neutral. However, because public good producers often have at least a slightly preferential access due to the mechanisms described above, this interference strategy will more likely lead to selection for resistance, albeit more weakly than in the case of traditional antibiotics. In addition, side-effects of a public good inhibitor may easily steer the selection towards resistance. A well-described example of such an inhibitor targeting the activity of the public good is gallium, an iron-mimic that irreversibly binds to the secreted siderophore pyoverdine, which renders secreted siderophores unable to bind iron (Ross-gillespie *et al*. 2014; Rezzoagli *et al*. 2018). Resistance against gallium in terms of recovery of a functional pyoverdine siderophore has not yet been described, probably due to evolving an increased contribution of alternative mechanisms for iron-uptake in *P. aeruginosa* (see ‘alternative resistance mechanisms’ section below).

(ii) In contrast, if the public good inhibitor directly targets the production of the public good, only resistant mutants that regained the production of the public good experience a fitness cost compared to sensitive mutants. Therefore, this strategy is not selection neutral but should select against resistance in case the previous screening steps indicated the targeted public good to be costly to produce and sufficiently shared with non-producers. However, in order to avoid side-effects that promote selection for resistance, this strategy requires high specificity. If a specific inhibitor is applied that only targets the public good without affecting coupled traits, stabilization mechanisms such as pleiotropy or policing will not apply since sensitive strains will still express the coupled trait. Difficulties associated with identifying such compounds might explain the lack of reports on inhibitors that directly target the production of public goods, although theoretically very promising.

(iii) Finally, the production of the public good can be targeted indirectly by interfering with its regulatory system. In ideal circumstances, this regulatory system is specific for the targeted public good or, if not, coordinates the production of public goods only. In case the public good is co-regulated with private traits essential for growth or survival in the environment under focus, this might strongly increase the cost of cheating and possibly lead to the enrichment of resistant mutants. However, in case the co-regulated traits are redundant in the relevant environment, resistance might imply a costly disadvantage and selection against resistance will occur (Allen *et al*. 2014). Our 5-step screening approach provides an easy proxy for assessing the suitability of upstream regulators as inhibitory targets. Indeed, if next to genes coding for the production of the public good, also upstream regulatory genes are identified during screening steps 2 and 3, targeting these regulatory mechanisms is likely evolutionarily robust. However, if the screening only revealed downstream genes, the regulatory networks likely include too many private genes in order to be evolution-proof.

An example of a public good inhibitor targeting the regulation of a public trait is flucytosine. This is a repurposed antifungal drug that inhibits the expression of the iron-starvation σ-factor PvdS in *P. aeruginosa*, thereby repressing the production of the pyoverdine siderophore among other virulence factors, such as PrpL protease and exotoxin A (Imperi *et al*. 2013). However, resistance evolution has been observed, probably due to pleiotropic and deleterious effects on private traits (Rezzoagli *et al*. 2018; Imperi *et al*. 2019). In contrast, a clear example of resistance proof activity of an inhibitor targeting regulation can be found in 2-cyclopentenyl-5-(4-chlorophenyl)-2-aminoimidazole (Dieltjens *et al*. 2020), inhibiting EPS production of *S*. Typhimurium by reducing the transcription of *csgD*, encoding the master regulator of EPS, and its regulon (Robijns *et al*. 2014).

In addition to the above examples of inhibitors targeting private regulation mechanisms, the target regulatory mechanism can also be public itself, as is the case for QS signalling. QS signalling that regulates the production of public behaviours has been the target of several public good inhibitors. A distinction can be made between signal-response inhibitors, which render susceptible cells signal-blind and impair the production of regulated phenotypes, and signal supply inhibitors, which reduce the signal levels (Allen *et al*. 2014). Signal-response inhibitors will impose similar selective pressures to those that have been described for inhibitors that directly target the production of the public good as long as the regulatory system is sufficiently specific. A promising example is the small molecule inhibitor savarin, which blocks AgrA-mediated QS public good regulation in *S. aureus* and did not select for resistance or tolerance in *in vitro* and *in vivo* serial passage evolution experiments for 10 days (Sully *et al*. 2014). Treatment with the synthetic QS-inhibitor furanone C-30, which suppresses both private and public traits did however invoke resistance (Maeda *et al*. 2012). The situation is markedly different for signal supply inhibitors, and this for two reasons. Firstly, since QS molecules are public themselves, signal supply inhibitors might even be evolutionarily robust in case the phenotypes they regulate are private themselves. Resistant cells will remain selection neutral since the sensitive cells will also produce the QS regulated trait in response to the signal secreted by the resistant cells. In case the signal would not be sufficiently shared, a second mechanism can additionally counteract resistance. Indeed, the quorum threshold will only be met when cooperators are present in high numbers, rendering resistance selection-neutral at low frequencies. This frequency-dependent concept of ‘co-operate if surrounded by co-operators’ has been demonstrated for QS inhibition in *P. aeruginosa* (Gerdt and Blackwell 2014b).

An extension to the idea of targeting public goods with inhibitors, is to directly introduce non-producers in a cooperative population. The subsequent invasion of these non-producing cheats in the population may destabilize public good production, potentially both reducing virulence and enhancing susceptibility to the immune system and antimicrobial treatment (Brown *et al*. 2009). For instance, QS cheats of *P. aeruginosa* could invade the population of QS bacteria in a mouse model, as such reducing virulence (Rumbaugh *et al*. 2009). Moreover, these non-producing cheats could be used as ‘Trojan horses’ when engineered in such a way that they carry additional useful alleles such as lethal toxins (Brown *et al*. 2009). However, this alternative strategy carries some risks as, in contrast to treatment with inhibitors, the public good production is not inhibited directly but rather gradually diminished over time by the invasion and successive enrichment of non-producers. Moreover, as pathogens constantly evolve, the non-producing cheat may differ in more than only public production, possibly altering the competition with the producers (Brown *et al*. 2009).

### STEP 5: Validation by experimental evolution in the presence of the inhibitor

Once a suitable drug is identified (step 4), a first validation step is to repeat the competition experiments from step 3, but now with a co-culture of a sensitive and resistant strain in the presence of the public good inhibitor. This validation experiment is necessary as competition between producers and non-producers cannot perfectly predict resistance evolution to the inhibitor due to differences in the level of inhibition between inhibitor and deletion mutant and potential side effects of the inhibitor. These competition experiments should also be performed in the appropriate conditions, as indicated in step 3. However, unlike with antibiotics where resistant cells are rather easily obtained by means of experimental evolution, identification of such a resistant strain against a public good inhibitor is challenging. After all, if the strategy is successful, resistant cells should be counter selected during experimental evolution. One possible solution is by high-throughput screening of separate single mutants from a mutant library in the presence of the inhibitor. Another option that avoids a mutant selection step is by analyzing resistance in a mutagenized population at the single cell level. Hereto, one could for example use a strain containing a fluorescent protein fused to the promoter of a target gene repressed by the inhibitor. Mutants resistant to the inhibitor via a mechanism upstream of the promotor, will express the target gene even in the presence of the inhibitor and will turn fluorescent. Single cell analysis by Fluorescence-Activated Cell Sorting or Microscopy and micromanipulation can then be used to isolate fluorescent cells from the pools of mutants. Alternatively, if the inhibitor targets the production of the public good, one might interfere with the regulation of the target gene to create mutants that are irresponsive to the inhibitor and consequently resistant.

As a final validation of the evolutionary robustness of the inhibitor, experimental evolution of a sensitive strain in the presence of the identified inhibitor should be performed. This experiment evaluates the risk for resistance development against the inhibitor and shows how the composition of the population evolves upon emergence of a single resistant cell. Moreover, if a resistant population would emerge, a thorough analysis of the resistant strains via phenotypic assays and genomic sequencing will allow to identify possible aspecific effects of the inhibitor. For example, as previously mentioned, resistance against the siderophore inhibitors flucytosine and 5-fluorouracil was selected for during experimental evolution because of aspecific effects of these inhibitors on private traits (Rezzoagli *et al*. 2018; Imperi *et al*. 2019). Furthermore, the 5-aryl-2-aminoimidazole-based EPS inhibitor reduces both EPS and planktonic growth at high concentrations, which resulted in the rapid evolution of resistance to these growth inhibitory effects at this concentration. However, since these off-target effects were not linked to the public good, the resistant strains were still susceptible to the public good inhibition. This indicates that aspecific effects of an inhibitor –in this case attributable to excessive dosing- do not necessarily lead to resistance against the public good inhibitory effect (Dieltjens *et al*. 2020). Similar to previous screening steps, this validation should also be performed in conditions closely resembling the infection site, in order to provide relevant selection pressures for resistance against both the specific and aspecific effects. The evolutionary robustness of the AgrA inhibitor savarin, for instance, was already validated to this degree via *in vivo* serial passage in mice (Sully *et al*. 2014).

Although the proposed screening approach in conditions relevant for the envisioned application should provide substantial valuable information on the evolutionary dynamics upon public good inhibitor treatment, we stress the importance of consequent *in vivo* and *in situ* validation of the results. Because many factors beyond those that can be simulated *in vitro* can play a part in resistance evolution*, in vivo* and *in situ* validation will still be required to reveal the ground truth. Hereto, the selection of a suitable model system is crucial. For several infections, different animal models of varying complexity are available, ranging from greater wax moth larvae (*Galleria mellonella*) and *Caenorhabditis elegans* worms to higher animals (Hapfelmeier and Hardt 2005; Jiminez *et al*. 2015). These lower animal models can serve as a valuable intermediate step to evaluate evolutionary robustness in more complex conditions, but due to high importance of closely mimicking the *in situ* environment, a validation in higher animals is advised. In these next preclinical and clinical stages of drug development following the here presented screening protocol, it remains of the utmost importance to complement the standard analyses with assays focusing on resistance evolution to obtain evolution-proof drugs. Moreover, the true relevance of stabilization mechanisms accounted for while screening can be validated during these final stages.

## Alternative resistance mechanisms

So far, our reflections on the expected consequences of public good-stabilizing mechanisms for resistance evolution rely on the assumption that the observed resistance mechanisms are private. However, antimicrobial resistance mechanisms can also be public in nature, as in the case of β-lactamases for instance. If a resistance mechanism against a public good inhibitor is public itself, both resistant and sensitive strains can produce the public good trait. In this case, resistance evolution will depend on the cost and exploitability of the emerged public resistance mechanism only, rather than on the targeted public good trait (Payne *et al*. 2013; Payne 2015).

Besides, the targeted phenotype could also be recovered via the development of an alternative bypassing mechanism. Indeed, in some environments where bacteria express multiple public goods simultaneously, the inhibition of one public good might lead to the production of another functionally related public good. Such evolution of a public bypassing mechanism was for instance observed under gallium treatment, where iron acquisition in a siderophore-independent manner was restored through an upregulation of the production of the redox-active extracellular toxin pyocyanin that can reduce ferric to ferrous iron (Rezzoagli *et al*. 2018). This upregulation of pyocyanin upon pyoverdine inhibition is also supported by previous research (García-Contreras *et al*. 2014; Ross-gillespie *et al*. 2014; O'Brien *et al*. 2017). Since this alternative public good produced by resistant variants is still prone to exploitation by sensitive non-producers, these resistant mutants are, however, still not expected to spread in the population (Ross-gillespie *et al*. 2014), maintaining the evolutionary robustness of the inhibitor. Besides restoring another public trait, there is also a risk for the development of an alternative private mechanism with a similar function as the targeted public good, which obstructs the evolutionary robustness of this public good inhibition strategy. For example, upregulation of the private *phu* system targeting the iron-rich heme molecule was observed in *P. aeruginosa* when cooperative iron acquisition via the siderophore pyoverdine was lost from the population (Andersen *et al*. 2018).

Finally, we note that public good genes themselves are also under continuous selection for novelty and specificity, potentially preventing the sensitive strains of benefiting from the public good and outcompeting the resistant strains (Meyer *et al*. 1997; Smith *et al*. 2005; Brown *et al*. 2009), even in those cases where resistance occurred via a recovery of the targeted public good itself.

## Conclusion and future perspectives

According to the sustainable development goals of the World Health Organization (WHO), ensuring healthy lives and promoting well-being at all ages is essential to achieve a more sustainable future. The increased development of antimicrobial resistance however threatens this prospect by rendering antibiotics ineffective and leaving us defenseless against infections that were readily treatable until today. This work contributes to the urgent search for alternative antimicrobial strategies that are less prone to resistance development. The proposed screening method is centered around public good targets that confer a collective benefit to other cells in the population. Our method highlights the importance of not only studying the effect of a targeted phenotype on disease, but also its cost, shareability and exploitability, as these are critical determinants of the evolutionary robustness of interference strategies. Moreover, the condition dependency of the exploitability of public goods stresses the value of characterizing and mimicking the *in situ* situation as closely as possible. New sensors and detection systems capable of measuring structure, density and frequency of pathogens *in situ* during the infection will therefore become crucial to develop and implement new antimicrobial strategies. Overall, the perspective of resistance-proof drugs is expected to contribute to a revitalization of industrial interest in developing new antimicrobial strategies. Interference with public goods even has potential beyond the microbiology field, since targeting cooperative phenotypes can ultimately be extended to other applications such as anti-cancer therapy or insecticides. For instance, the tumor micro environment surrounding tumor cells resembles the EPS matrix surrounding microbes in a biofilm (Zhong *et al*. 2020), while communication via pheromones in social insects resembles microbial communication via quorum sensing (Seeley 1995). Such presumed cooperative phenotypes in higher organisms may also be susceptible to exploitation by non-cooperative cheaters in certain cases (Charlotte Jandér and Allen Herre 2010; Riehl and Frederickson 2016).

## Supplementary Material

fuac019_Supplemental_Files
