# Supplementary material for: Evolution-proof inhibitors of public good cooperation: a screening strategy inspired by social evolution theory
Source: FEMS Microbiol Rev. 2022 Jun 8;46(5):fuac019. doi: 10.1093/femsre/fuac019 (PMC9616471; doi:10.1093/femsre/fuac019)
Supplement: fuac019_Supplemental_Files [file fuac019_supplemental_files.zip › Supplementary_information-141221_Finaal_(1).docx]

Evolution-proof inhibitors of public good cooperation: a screening strategy inspired by social evolution theory

Supplementary information

Lissens *et al.*

**Supplementary Table S1: The exploitability of virulence factors previously identified as public goods is strongly dependent on environmental conditions.**

| **Species** | **Public good** | **Conditions supporting exploitability** | **Conditions counteracting exploitability** | |  |  |
| --- | --- | --- | --- | --- | --- | --- |
| **Virulence** | | | | |  |  |
| **Toxins:** Molecules damaging host tissue to promote infection and disease ^1^ | | | | |  |  |
| *B. thuringiensis* | Crystal toxin | High initial cell densities ^2^ | Low initial cell densities ^2^ | |  |  |
|  |  | High producer frequencies ^2^ | Low producer frequencies ^2^ | |  |  |
| *E. coli* | Shiga toxin | High producer frequencies ^3^ | Low producer frequencies ^3^ | |  |  |
| **Invasion effectors:** Virulence factors that enable bacteria to invade host tissue ^4^ | | | | |  |  |
| *S. enterica* | T3SS-1 | Intermediate producer frequencies ^4^ |  | |  |  |
| *P. aeruginosa* | T3SS | High producer frequencies ^5^ | Low producer frequencies ^5^ | |  |  |
| **Tolerance** | | | | |  |  |
| **Adhesion factors and biofilm matrix components:** Extracellular proteins and polymers mediating the attachment of cells to one another and to surfaces ^6^ and providing structure and protection against environmental conditions ^7,8^ | | | | |  |  |
| *B. subtilis* | EPS | High initial cell densities ^9^ | Low initial cell densities ^9^ | |  |  |
| *P. aeruginosa* | PSL |  | Structured biofilms ^10^ | |  |  |
|  | PEL | Structurally heterogeneous colony environment ^11^ | Structurally homogeneous pellicle environment ^11^ | |  |  |
| *P. fluorescens* | Polymer secretions | Spatially heterogeneous microcosms ^12^ | Structured colonies ^13^ | |  |  |
| *S. enterica* | EPS | Structured biofilms ^14^, both at high and low producer frequencies ^15^ |  | |  |  |
| *V. cholera* | EPS |  | Structured biofilms in flow environment ^6,16^ | |  |  |
|  | RbmA |  | Structured biofilms ^17^ | |  |  |
|  | Bap1 | Structured biofilms ^18^ |  | |  |  |
|  | VPS |  | Structured biofilms ^18^ | |  |  |
| **Collective resistance mechanisms:** Resistance mechanisms allowing the survival of non-resistant bacteria during antimicrobial treatment ^19^ | | | | |  |  |
| *E. coli* | β- lactamase | Structured colony biofilms ^20,21^ and, to a lesser extent, well-mixed liquid cultures ^20–22^ |  | |  |  |
|  |  | High producer frequencies^20,22^ | Low producer frequencies ^22^ | |  |  |
|  |  | High initial cell densities ^20^ | Low initial cell densities ^20^ | |  |  |
|  |  | Low initial antibiotic concentration below the MIC value ^23^ and fast detoxification ^24^ | High initial antibiotic concentration exceeding the MIC value ^23^ and slow detoxification ^24^ | |  |  |
| *P. aeruginosa* | β- lactamase | Structured colony biofilms ^25^ and a flow cell biofilm model ^26^ | Well-mixed liquid cultures ^25,26^ | |  |  |
|  |  | Intermediate producer frequencies ^26^ | Low producer frequencies ^26^ | |  |  |
|  |  | High initial cell densities and mixing bacterial colonies, resulting in genetically mixed colonies ^25^ | Low initial cell densities, resulting in large clonal patches within colonies ^25^ | |  |  |
|  |  | Low initial antibiotic concentration below the MIC value, resulting in cell elongation of susceptible cells ^25^ | High initial antibiotic concentration exceeding the MIC value ^25^ | |  |  |
|  | MexAB-OprM Efflux pump | Structured flow cell biofilm model ^26^ | Well-mixed liquid cultures ^26^ | |  |  |
|  |  | Intermediate producer frequencies ^26^ | Low producer frequencies ^26^ | |  |  |
| **Nutrient availability** | | | | | | |
| **Siderophores**: Iron-scavenging molecules, forming soluble Fe^3+^ complexes and allowing iron uptake through the cell membrane via specific receptors ^27,28^ | | | | | | |
| *B.*  *cenocepacia* | ornibactin |  | | Static and well-mixed liquid cultures ^29^ | | |
|  |  |  | | High and low initial cell densities ^29^ | | |
|  |  |  | | High and low producer frequencies ^29^ | | |
| *B.*  *cenocepacia* | pyochelin | Static and, to a lesser extent, well-mixed liquid cultures ^29^ | |  | | |
|  |  | High and low initial cell densities ^29^ | |  | | |
|  |  | Low producer frequencies ^29^ | | High producer frequencies ^29^ | | |
| *E. coli* | Enterochelin | High initial cell densities ^30^ | | Low initial cell densities ^30^ | | |
| *P. aeruginosa* | Pyochelin | Strong and moderate iron-limitation ^31^ | |  | | |
|  | Pyoverdin | Strong iron-limitation ^31,32^ | | Low ^32^ and moderate ^31^ iron-limitation | | |
|  |  | Low relatedness and local competition ^33^ | | High relatedness and global competition ^33^ | | |
|  |  | Well-mixed liquid cultures with low spatial structure ^32,34^ and colonies on soft surfaces ^27^ | | Viscous static liquid cultures with high spatial structure ^32,34^ and colonies on hard surfaces ^27^ | | |
|  |  | High initial cell densities ^35^ | | Low initial cell densities ^35^ | | |
|  |  | High producer frequencies ^36^ | | Low producer frequencies ^36^ | | |
|  |  | Resources needed to make the siderophore are growth-limiting ^37^ | | Resources needed to make the siderophore are in relative excess ^37^ | | |
|  |  | In lag and early exponential growth phase ^38^ | | During late exponential or stationary phase ^38^ | | |
| *P. fluorescens* | Pyoverdin | Strong iron-limitation ^28^ | | Low and moderate iron-limitation ^28^ | | |
|  |  | High producer frequencies ^28^ | | Low producer frequencies ^28^ | | |
| **Extracellular enzymes**: Enzymes mediating digestion of complex molecules (e.g. proteins, polymers) into smaller molecules (e.g. polypeptides, monosaccharides) ^39,40^ | | | | | | |
| *P. aeruginosa* | Proteases such as elastase (regulated by QS) | Conditions requiring elastase ^41–44^ | | Conditions not requiring elastase ^41,43,44^ | | |
|  |  | Well-mixed liquid ^45^ and viscous ^44^ cultures, as well as flow cell biofilms and biofilms on plastic beads ^46^ | |  | | |
|  |  | In intubated patients ^47^ | |  | | |
|  |  | High producer frequencies ^45,48^ | | Low producer frequencies ^45,48^ | | |
|  |  | Constitutive production ^49^ | | Facultative regulation through QS ^49^ | | |
|  |  | In absence of cyanide producers ^50^ | | In presence of cyanide producers ^50^ | | |
| *S. cerevisiae* | Invertase | Structured agar plate ^51^ and well-mixed liquid cultures ^39,52^ | | Static liquid cultures with high spatial structure ^52^ | | |
|  |  | High initial cell densities ^51^ | | Low initial cell densities ^51^ | | |
|  |  | High producer frequencies ^39,52^ | | Low producer frequencies ^39,52^ | | |
| *V. cholera* | Chitinase | Well-mixed liquid cultures ^53^ | | Thick biofilms and fluid flow ^53^ | | |
|  |  | High producer frequencies in static liquid cultures ^53^ | | Low producer frequencies in static liquid cultures ^53^ | | |
| **Motility** | | | | | | |
| **Biosurfactants**: Compounds mediating swarming motility over surfaces ^54^ | | | | | | |
| *P. aeruginosa* | Rhamnolipid | Constitutive production ^54^ | | Growth-limiting conditions due to a nutrient which is not needed for rhamnolipid production ^54^ | | |
| \| **Competition** \| \| --- \| \| **Bacteriocins:** Antimicrobial molecules produced by microbes to decrease the fitness of other bacterial species ^55^ \| | | | | | | |
| *E. coli* | Colicin | Well-mixed liquid cultures ^55^ | | Structured soft agar matrix ^55^ | | |
|  |  | Low producer frequencies ^55,56^ | | High producer frequencies ^55,56^ | | |
| **Communication** | | | | | |  |
| **Quorum sensing signals:** Small diffusible signalling molecules mediating bacterial cell-to-cell communication ^48^ | | | | | |  |
| *P. aeruginosa* | LasI signal | Well-mixed liquid cultures ^57^ | | Solid agar, reducing diffusion ^57^ | |  |
|  |  | In acute burn and chronic wound mouse models ^58^ | |  | |  |
|  |  | High producer frequencies ^48,57,58^ | | Low producer frequencies ^48,57,58^ | |  |

**Reference list**

1. Henkel, J. S., Baldwin, M. R. & Barbieri, J. T. Toxins from bacteria. *EXS* **100,** 1–29 (2010).

2. Raymond, B., West, S. A., Griffin, A. S. & Bonsall, M. B. The Dynamics of Cooperative Bacterial Virulence in the Field. *Science* **337,** 85–89 (2012).

3. Aijaz, I. & Koudelka, G. B. Cheating, facilitation and cooperation regulate the effectiveness of phage-encoded exotoxins as antipredator molecules. *MicrobiologyOpen* **8,** e636 (2019).

4. Diard, M. *et al.* Stabilization of cooperative virulence by the expression of an avirulent phenotype. *Nature* **494,** 353–356 (2013).

5. Czechowska, K., Mckeithen-mead, S., Al, K. & Kazmierczak, B. I. Cheating by type 3 secretion system-negative Pseudomonas aeruginosa during pulmonary infection. *PNAS* **111,** 7801–7806 (2014).

6. Schluter, J., Nadell, C. D., Bassler, B. L. & Foster, K. R. Adhesion as a weapon in microbial competition. *ISME J.* **9,** 139–149 (2015).

7. Mah, T. F. Biofilm-specific antibiotic resistance. *Future Microbiol.* **7,** 1061–1072 (2012).

8. Hall-Stoodley, L., Costerton, J. W. & Stoodley, P. Bacterial biofilms: From the natural environment to infectious diseases. *Nat. Rev. Microbiol.* **2,** 95–108 (2004).

9. Van Gestel, J., Weissing, F. J., Kuipers, O. P. & Kovács, Á. T. Density of founder cells affects spatial pattern formation and cooperation in Bacillus subtilis biofilms. *ISME J.* **8,** 2069–2079 (2014).

10. Irie, Y. *et al.* The Pseudomonas aeruginosa PSL Polysaccharide Is a Social but Noncheatable Trait in Biofilms. *MBio* **8,** e00374-17 (2017).

11. Madsen, J. S. *et al.* Facultative Control of Matrix Production Optimizes Competitive Fitness in Pseudomonas aeruginosa PA14 Biofilm Models. *Appl. Environ. Microbiol.* **81,** 8414–8426 (2015).

12. Rainey, P. B. & Rainey, K. Evolution of cooperation and conflict in experimental bacterial populations. *Nature* **425,** 72–74 (2003).

13. Kim, W., Racimo, F., Schluter, J., Levy, S. B. & Foster, K. R. Importance of positioning for microbial evolution. *Proc. Natl. Acad. Sci.* **111,** E1639–E1647 (2014).

14. Srinandan, C. S., Elango, M., Gnanadhas, D. P. & Chakravortty, D. Infiltration of matrix-non-producers weakens the salmonella biofilm and impairs its antimicrobial tolerance and pathogenicity. *Front. Microbiol.* **6,** 1468 (2015).

15. Dieltjens, L. *et al.* Inhibiting bacterial cooperation is an evolutionarily robust anti-biofilm strategy. *Nat. Commun.* **11,** 107 (2020).

16. Nadell, C. D. & Bassler, B. L. A fitness trade-off between local competition and dispersal in Vibrio cholerae biofilms. *PNAS* **108,** 14181–14185 (2011).

17. Nadell, C. D., Drescher, K., Wingreen, N. S. & Bassler, B. L. Extracellular matrix structure governs invasion resistance in bacterial biofilms. *ISME J.* **9,** 1700–1709 (2015).

18. Absalon, C., Dellen, K. Van & Watnick, P. I. A Communal Bacterial Adhesin Anchors Biofilm and Bystander Cells to Surfaces. *PLoS Pathog.* **7,** e1002210 (2011).

19. Vega, N. M. & Gore, J. Collective antibiotic resistance: Mechanisms and implications. *Curr. Opin. Microbiol.* **21,** 28–34 (2014).

20. Domingues, I. L., Gama, J. A., Carvalho, L. M. & Dionisio, F. Social behaviour involving drug resistance: the role of initial density, initial frequency and population structure in shaping the effect of antibiotic resistance as a public good. *Heredity (Edinb).* **119,** 295–301 (2017).

21. Amanatidou, E. *et al.* Biofilms facilitate cheating and social exploitation of β-lactam resistance in Escherichia coli. *npj Biofilms Microbiomes* **5,** 36 (2019).

22. Yurtsev, E. A., Chao, H. X., Datta, M. S., Artemova, T. & Gore, J. Bacterial cheating drives the population dynamics of cooperative antibiotic resistance plasmids. *Mol. Syst. Biol.* **9,** 683 (2013).

23. Bottery, M. J., Wood, A. J. & Brockhurst, A. Selective Conditions for a Multidrug Resistance Plasmid Depend on the Sociality of Antibiotic Resistance. *Antimicrob Agents Chemother.* **60,** 2524–2527 (2016).

24. Medaney, F., Dimitriu, T., Ellis, R. J. & Raymond, B. Live to cheat another day: bacterial dormancy facilitates the social exploitation of β-lactamases. *ISME J.* **10,** 778–787 (2016).

25. Frost, I. *et al.* Cooperation, competition and antibiotic resistance in bacterial colonies. *ISME J.* **12,** 1582–1593 (2018).

26. Rojo-Molinero, E., Macià, M. D. & Oliver, A. Social Behavior of Antibiotic Resistant Mutants Within Pseudomonas aeruginosa Biofilm Communities. *Front. Microbiol.* **10,** 570 (2019).

27. Weigert, M. & Kümmerli, R. The physical boundaries of public goods cooperation between surface-attached bacterial cells. *Proc R Soc B* **284,** 20170631 (2017).

28. Zhang, X. & Rainey, P. B. Exploring the sociobiology of pyoverdin-producing Pseudomonas. *Evolution* **67,** 3161–3174 (2013).

29. Sathe, S., Mathew, A., Agnoli, K., Eberl, L. & Kümmerli, R. Genetic architecture constrains exploitation of siderophore cooperation in the bacterium Burkholderia cenocepacia. *Evol. Lett.* **3,** 610–622 (2019).

30. Scholz, R. L. & Greenberg, E. P. Sociality in Escherichia coli: Enterochelin Is a Private Good at Low Cell Density and Can Be Shared at High Cell Density. *J. Bacteriol.* **197,** 2122–2128 (2015).

31. Ross-Gillespie, A., Dumas, Z. & Kümmerli, R. Evolutionary dynamics of interlinked public goods traits: an experimental study of siderophore production in Pseudomonas aeruginosa. *J. Evol. Biol.* **28,** 29–39 (2015).

32. Leinweber, A., Fredrik Inglis, R. & Kümmerli, R. Cheating fosters species co-existence in well-mixed bacterial communities. *ISME J.* **11,** 1179–1188 (2017).

33. Griffin, A. S., West, S. A. & Buckling, A. Cooperation and competition in pathogenic bacteria. *Nature* **430,** 1024–1027 (2004).

34. Kümmerli, R., Griffin, A. S., West, S. A., Buckling, A. & Harrison, F. Viscous medium promotes cooperation in the pathogenic bacterium Pseudomonas aeruginosa. *Proc R Soc B* **276,** 3531–3538 (2009).

35. Ross-gillespie, A., Gardner, A., Buckling, A., West, S. A. & Griffin, A. S. Density dependence and cooperation: theory and a test with bacteria. *Evolution* **63,** 2315–2325 (2009).

36. Ross-gillespie, A., Gardner, A., West, S. A. & Griffin, A. S. Frequency Dependence and Cooperation: Theory and a Test with Bacteria. *Am. Nat.* **170,** 331–342 (2007).

37. Sexton, D. J. & Schuster, M. Nutrient limitation determines the fitness of cheaters in bacterial siderophore cooperation. *Nat. Commun.* **8,** 230 (2017).

38. Ghoul, M. *et al.* Pyoverdin cheats fail to invade bacterial populations in stationary phase. *J. Evol. Biol.* **29,** 1728–1736 (2016).

39. Gore, J., Youk, H. & van Oudenaarden, A. Snowdrift game dynamics and facultative cheating in yeast. *Nature* **459,** 253–256 (2009).

40. Özkaya, Ö., Xavier, K. B., Dionisio, F. & Balbontín, R. Maintenance of microbial cooperation mediated by public goods in single- and multiple-trait scenarios. *J. Bacteriol.* **199,** e00297-17 (2017).

41. Özkaya, Ö., Balbontín, R., Gordo, I. & Xavier, K. B. Cheating on Cheaters Stabilizes Cooperation in Pseudomonas aeruginosa. *Curr. Biol.* **28,** 2070–2080 (2018).

42. Sandoz, K. M., Mitzimberg, S. M. & Schuster, M. Social cheating in Pseudomonas aeruginosa quorum sensing. *PNAS* **104,** 15876–15881 (2007).

43. Mellbye, B. & Schuster, M. The Sociomicrobiology of Antivirulence Drug Resistance: a Proof of Concept. *MBio* **2,** 3–6 (2011).

44. Gerdt, J. P. & Blackwell, H. E. Competition Studies Confirm Two Major Barriers That Can Preclude the Spread of Resistance to Quorum-Sensing Inhibitors in Bacteria. *ACS Chem. Biol.* **9,** 2291–2299 (2014).

45. Wilder, C. N., Diggle, S. P. & Schuster, M. Cooperation and cheating in Pseudomonas aeruginosa: the roles of the las, rhl and pqs quorum-sensing systems. *ISME J.* **5,** 1332–1343 (2011).

46. Popat, R. *et al.* Quorum-sensing and cheating in bacterial biofilms. *Proc R Soc B* **279,** 4765–4771 (2012).

47. Köhler, T., Perron, G. G., Buckling, A. & van Delden, C. Quorum Sensing Inhibition Selects for Virulence and Cooperation in Pseudomonas aeruginosa. *PLoS Pathog.* **6,** e1000883 (2010).

48. Diggle, S. P., Griffin, A. S., Campbell, G. S. & West, S. A. Cooperation and conflict in quorum-sensing bacterial populations. *Nature* **450,** 411–414 (2007).

49. Allen, R. C., Mcnally, L., Popat, R. & Brown, S. P. Quorum sensing protects bacterial co-operation from exploitation by cheats. *ISME J.* **10,** 1706–1716 (2016).

50. Wang, M., Schaefer, A. L., Dandekar, A. A. & Greenberg, E. P. Quorum sensing and policing of Pseudomonas aeruginosa social cheaters. *PNAS* **112,** 2187–2191 (2015).

51. Greig, D. & Travisano, M. The Prisoner’s Dilemma and polymorphism in yeast SUC genes. *Proc. R. Soc. London. Ser. B Biol. Sci.* **271,** S25–S26 (2004).

52. Maclean, R. C., Fuentes-hernandez, A., Greig, D., Hurst, L. D. & Gudelj, I. A Mixture of ‘“Cheats”’ and ‘“Co-Operators”’ Can Enable Maximal Group Benefit. *PLoS Biol.* **8,** e1000486 (2010).

53. Drescher, K., Nadell, C. D., Stone, H. A., Wingreen, N. S. & Bassler, B. L. Solutions to the public goods dilemma in bacterial biofilms. *Curr. Biol.* **24,** 50–55 (2014).

54. Xavier, J. B., Kim, W. & Foster, K. R. A molecular mechanism that stabilizes cooperative secretions in Pseudomonas aeruginosa. *Mol. Microbiol.* **79,** 166–179 (2011).

55. Chao, L. & Levin, B. R. Structured habitats and the evolution of anticompetitor toxins in bacteria. *Proc Natl Acad Sci U S A* **78,** 6324–6328 (1981).

56. Adams, J., Kinney, T., Thompson, S., Rubin, L. & Helling, R. B. Frequency-dependent selection for plasmid-containing cells of Escherichia coli. *Genetics* **91,** 627–637 (1979).

57. Mund, A., Diggle, S. P. & Harrison, F. The Fitness of Pseudomonas aeruginosa Quorum Sensing Signal Cheats Is Influenced by the Diffusivity of the Environment. *MBio* **8,** e00353-17 (2017).

58. Rumbaugh, K. P. *et al.* Quorum Sensing and the Social Evolution of Bacterial Virulence. *Curr. Biol.* **19,** 341–345 (2009).
