# Supplementary material for: Evolution-proof inhibitors of public good cooperation: a screening strategy inspired by social evolution theory
Source: FEMS Microbiol Rev. 2022 Jun 8;46(5):fuac019. doi: 10.1093/femsre/fuac019 (PMC9616471; doi:10.1093/femsre/fuac019)
Supplement: fuac019_Supplemental_Files [file fuac019_supplemental_files.zip › Supplementary_information-141221_Finaal_(1).pdf]

Lissens *et al.*

Evolution-proof inhibitors of public good cooperation: a screening strategy inspired by social evolution theory

Supplementary information

Lissens *et al.*

Supplementary Table S1: The exploitability of virulence factors previously identified as public goods is strongly dependent on environmental conditions.

Species	Public good	Conditions supporting exploitability	Conditions counteracting exploitability
**Virulence**			
**Toxins:** Molecules damaging host tissue to promote infection and disease [1]			
B. thuringiensis	Crystal toxin	High initial cell densities [2]<br>High producer frequencies [2]	Low initial cell densities [2]<br>Low producer frequencies [2]
E. coli	Shiga toxin	High producer frequencies [3]	Low producer frequencies [3]
**Invasion effectors:** Virulence factors that enable bacteria to invade host tissue [4]			
S. enterica	T3SS-1	Intermediate producer frequencies [4]	
P. aeruginosa	T3SS	High producer frequencies [5]	Low producer frequencies [5]
**Tolerance**			
**Adhesion factors and biofilm matrix components:** Extracellular proteins and polymers mediating the attachment of cells to one another and to surfaces [6] and providing structure and protection against environmental conditions [7,8]			
B. subtilis	EPS	High initial cell densities [9]	Low initial cell densities [9]
P. aeruginosa	PSL		Structured biofilms [10]
	PEL	Structurally heterogeneous colony environment [11]	Structurally homogeneous pellicle environment [11]
P. fluorescens	Polymer secretions	Spatially heterogeneous microcosms [12]	Structured colonies [13]
S. enterica	EPS	Structured biofilms [14], both at high and low producer frequencies [15]	
V. cholera	EPS		Structured biofilms in flow environment [6,16]
	RbmA		Structured biofilms [17]
	Bap1	Structured biofilms [18]	
	VPS		Structured biofilms [18]
**Collective resistance mechanisms:** Resistance mechanisms allowing the survival of non-resistant bacteria during antimicrobial treatment [19]			
E. coli	β- lactamase	Structured colony biofilms [20,21] and, to a lesser extent, well-mixed liquid cultures [20–22]<br>High producer frequencies[20,22]<br>High initial cell densities [20]<br>Low initial antibiotic concentration below the MIC value [23] and fast detoxification [24]	Low producer frequencies [22]<br>Low initial cell densities [20]<br>High initial antibiotic concentration exceeding the MIC value [23] and slow detoxification [24]
P. aeruginosa	β- lactamase	Structured colony biofilms [25] and a flow cell biofilm model [26]<br>Intermediate producer frequencies [26]<br>High initial cell densities and mixing bacterial colonies, resulting in genetically mixed colonies [25]<br>Low initial antibiotic concentration below the MIC value, resulting in cell elongation of susceptible cells [25]	Well-mixed liquid cultures [25,26]<br>Low producer frequencies [26]<br>Low initial cell densities, resulting in large clonal patches within colonies [25]<br>High initial antibiotic concentration exceeding the MIC value [25]
	MexAB-OprM Efflux pump	Structured flow cell biofilm model [26]<br>Intermediate producer frequencies [26]	Well-mixed liquid cultures [26]<br>Low producer frequencies [26]
**Nutrient availability**			
**Siderophores**: Iron-scavenging molecules, forming soluble Fe^{3+} complexes and allowing iron uptake through the cell membrane via specific receptors [27,28]			
B. cenocepacia	ornibactin		Static and well-mixed liquid cultures [29]<br>High and low initial cell densities [29]<br>High and low producer frequencies [29]

B. cenocepacia	pyochelin	Static and, to a lesser extent, well-mixed liquid cultures [29]<br>High and low initial cell densities [29]<br>Low producer frequencies [29]	High producer frequencies [29]
E. coli	Enterochelin	High initial cell densities [30]	Low initial cell densities [30]
P. aeruginosa	Pyochelin	Strong and moderate iron-limitation [31]	
	Pyoverdin	Strong iron-limitation [31,32]<br>Low relatedness and local competition [33]<br>Well-mixed liquid cultures with low spatial structure [32,34] and colonies on soft surfaces [27]<br>High initial cell densities [35]<br>High producer frequencies [36]<br>Resources needed to make the siderophore are growth-limiting [37]<br>In lag and early exponential growth phase [38]	Low [32] and moderate [31] iron-limitation<br>High relatedness and global competition [33]<br>Viscous static liquid cultures with high spatial structure [32,34] and colonies on hard surfaces [27]<br>Low initial cell densities [35]<br>Low producer frequencies [36]<br>Resources needed to make the siderophore are in relative excess [37]<br>During late exponential or stationary phase [38]
P. fluorescens	Pyoverdin	Strong iron-limitation [28]<br>High producer frequencies [28]	Low and moderate iron-limitation [28]<br>Low producer frequencies [28]
**Extracellular enzymes**: Enzymes mediating digestion of complex molecules (e.g. proteins, polymers) into smaller molecules (e.g. polypeptides, monosaccharides) [39,40]			
P. aeruginosa	Proteases such as elastase (regulated by QS)	Conditions requiring elastase [41–44]<br>Well-mixed liquid [45] and viscous [44] cultures, as well as flow cell biofilms and biofilms on plastic beads [46]<br>In intubated patients [47]<br>High producer frequencies [45,48]<br>Constitutive production [49]<br>In absence of cyanide producers [50]	Conditions not requiring elastase [41,43,44]<br>Low producer frequencies [45,48]<br>Facultative regulation through QS [49]<br>In presence of cyanide producers [50]
S. cerevisiae	Invertase	Structured agar plate [51] and well-mixed liquid cultures [39,52]<br>High initial cell densities [51]<br>High producer frequencies [39,52]	Static liquid cultures with high spatial structure [52]<br>Low initial cell densities [51]<br>Low producer frequencies [39,52]
V. cholera	Chitinase	Well-mixed liquid cultures [53]<br>High producer frequencies in static liquid cultures [53]	Thick biofilms and fluid flow [53]<br>Low producer frequencies in static liquid cultures [53]
**Motility**			
**Biosurfactants**: Compounds mediating swarming motility over surfaces [54]			
P. aeruginosa	Rhamnolipid	Constitutive production [54]	Growth-limiting conditions due to a nutrient which is not needed for rhamnolipid production [54]
**Competition**			
**Bacteriocins:** Antimicrobial molecules produced by microbes to decrease the fitness of other bacterial species [55]			
E. coli	Colicin	Well-mixed liquid cultures [55]<br>Low producer frequencies [55,56]	Structured soft agar matrix [55]<br>High producer frequencies [55,56]
**Communication**			
**Quorum sensing signals:** Small diffusible signalling molecules mediating bacterial cell-to-cell communication [48]			
P. aeruginosa	LasI signal	Well-mixed liquid cultures [57]<br>In acute burn and chronic wound mouse models [58]<br>High producer frequencies [48,57,58]	Solid agar, reducing diffusion [57]<br>Low producer frequencies [48,57,58]

Reference list

1. Henkel, J. S., Baldwin, M. R. & Barbieri, J. T. Toxins from bacteria. *EXS* **100,** 1–29 (2010).

2. Raymond, B., West, S. A., Griffin, A. S. & Bonsall, M. B. The Dynamics of Cooperative Bacterial Virulence in the Field. *Science* **337,** 85–89 (2012).

3. Aijaz, I. & Koudelka, G. B. Cheating, facilitation and cooperation regulate the effectiveness of phage-encoded exotoxins as antipredator molecules. *MicrobiologyOpen* **8,** e636 (2019).

4. Diard, M. *et al.* Stabilization of cooperative virulence by the expression of an avirulent phenotype. *Nature* **494,** 353–356 (2013).

5. Czechowska, K., Mckeithen-mead, S., Al, K. & Kazmierczak, B. I. Cheating by type 3 secretion system-negative Pseudomonas aeruginosa during pulmonary infection. *PNAS* **111,** 7801–7806 (2014).

6. Schluter, J., Nadell, C. D., Bassler, B. L. & Foster, K. R. Adhesion as a weapon in microbial competition. *ISME J.* **9,** 139–149 (2015).

7. Mah, T. F. Biofilm-specific antibiotic resistance. *Future Microbiol.* **7,** 1061–1072 (2012).

8. Hall-Stoodley, L., Costerton, J. W. & Stoodley, P. Bacterial biofilms: From the natural environment to infectious diseases. *Nat. Rev. Microbiol.* **2,** 95–108 (2004).

9. Van Gestel, J., Weissing, F. J., Kuipers, O. P. & Kovács, Á. T. Density of founder cells affects spatial pattern formation and cooperation in Bacillus subtilis biofilms. *ISME J.* **8,** 2069–2079 (2014).

10. Irie, Y. *et al.* The Pseudomonas aeruginosa PSL Polysaccharide Is a Social but Noncheatable Trait in Biofilms. *MBio* **8,** e00374-17 (2017).

11. Madsen, J. S. *et al.* Facultative Control of Matrix Production Optimizes Competitive Fitness in Pseudomonas aeruginosa PA14 Biofilm Models. *Appl. Environ. Microbiol.* **81,** 8414–8426 (2015).

12. Rainey, P. B. & Rainey, K. Evolution of cooperation and conflict in experimental bacterial populations. *Nature* **425,** 72–74 (2003).

13. Kim, W., Racimo, F., Schluter, J., Levy, S. B. & Foster, K. R. Importance of positioning for microbial evolution. *Proc. Natl. Acad. Sci.* **111,** E1639–E1647 (2014).

14. Srinandan, C. S., Elango, M., Gnanadhas, D. P. & Chakravortty, D. Infiltration of matrix-non-producers weakens the salmonella biofilm and impairs its antimicrobial tolerance and pathogenicity. *Front. Microbiol.* **6,** 1468 (2015).

15. Dieltjens, L. *et al.* Inhibiting bacterial cooperation is an evolutionarily robust anti-biofilm strategy. *Nat. Commun.* **11,** 107 (2020).

16. Nadell, C. D. & Bassler, B. L. A fitness trade-off between local competition and dispersal in Vibrio cholerae biofilms. *PNAS* **108,** 14181–14185 (2011).

17. Nadell, C. D., Drescher, K., Wingreen, N. S. & Bassler, B. L. Extracellular matrix structure governs invasion resistance in bacterial biofilms. *ISME J.* **9,** 1700–1709 (2015).

18. Absalon, C., Dellen, K. Van & Watnick, P. I. A Communal Bacterial Adhesin Anchors Biofilm and Bystander Cells to Surfaces. *PLoS Pathog.* **7,** e1002210 (2011).

19. Vega, N. M. & Gore, J. Collective antibiotic resistance: Mechanisms and implications. *Curr. Opin. Microbiol.* **21,** 28–34 (2014).

20. Domingues, I. L., Gama, J. A., Carvalho, L. M. & Dionisio, F. Social behaviour involving drug resistance: the role of initial density, initial frequency and population structure in shaping the effect of antibiotic resistance as a public good. *Heredity (Edinb).* **119,** 295–301 (2017).

21. Amanatidou, E. *et al.* Biofilms facilitate cheating and social exploitation of β-lactam resistance in Escherichia coli. *npj Biofilms Microbiomes* **5,** 36 (2019).

22. Yurtsev, E. A., Chao, H. X., Datta, M. S., Artemova, T. & Gore, J. Bacterial cheating drives the population dynamics of cooperative antibiotic resistance plasmids. *Mol. Syst. Biol.* **9,** 683 (2013).

23. Bottery, M. J., Wood, A. J. & Brockhurst, A. Selective Conditions for a Multidrug Resistance Plasmid Depend on the Sociality of Antibiotic Resistance. *Antimicrob Agents Chemother.* **60,** 2524–2527 (2016).

24. Medaney, F., Dimitriu, T., Ellis, R. J. & Raymond, B. Live to cheat another day: bacterial dormancy facilitates the social exploitation of β-lactamases. *ISME J.* **10,** 778–787 (2016).

25. Frost, I. *et al.* Cooperation, competition and antibiotic resistance in bacterial colonies. *ISME J.* **12,** 1582–1593 (2018).

26. Rojo-Molinero, E., Macià, M. D. & Oliver, A. Social Behavior of Antibiotic Resistant Mutants Within Pseudomonas aeruginosa Biofilm Communities. *Front. Microbiol.* **10,** 570 (2019).

27. Weigert, M. & Kümmerli, R. The physical boundaries of public goods cooperation between surface-attached bacterial cells. *Proc R Soc B* **284,** 20170631 (2017).

28. Zhang, X. & Rainey, P. B. Exploring the sociobiology of pyoverdin-producing Pseudomonas. *Evolution* **67,** 3161–3174 (2013).

29. Sathe, S., Mathew, A., Agnoli, K., Eberl, L. & Kümmerli, R. Genetic architecture constrains exploitation of siderophore cooperation in the bacterium Burkholderia cenocepacia. *Evol. Lett.* **3,** 610–622 (2019).

30. Scholz, R. L. & Greenberg, E. P. Sociality in Escherichia coli: Enterochelin Is a Private Good at Low Cell Density and Can Be Shared at High Cell Density. *J. Bacteriol.* **197,** 2122–2128 (2015).

31. Ross-Gillespie, A., Dumas, Z. & Kümmerli, R. Evolutionary dynamics of interlinked public goods traits: an experimental study of siderophore production in Pseudomonas aeruginosa. *J. Evol. Biol.* **28,** 29–39 (2015).

32. Leinweber, A., Fredrik Inglis, R. & Kümmerli, R. Cheating fosters species co-existence in well-mixed bacterial communities. *ISME J.* **11,** 1179–1188 (2017).

33. Griffin, A. S., West, S. A. & Buckling, A. Cooperation and competition in pathogenic bacteria. *Nature* **430,** 1024–1027 (2004).

34. Kümmerli, R., Griffin, A. S., West, S. A., Buckling, A. & Harrison, F. Viscous medium promotes cooperation in the pathogenic bacterium Pseudomonas aeruginosa. *Proc R Soc B* **276,** 3531–3538 (2009).

35. Ross-gillespie, A., Gardner, A., Buckling, A., West, S. A. & Griffin, A. S. Density dependence and cooperation: theory and a test with bacteria. *Evolution* **63,** 2315–2325 (2009).

36. Ross-gillespie, A., Gardner, A., West, S. A. & Griffin, A. S. Frequency Dependence and Cooperation: Theory and a Test with Bacteria. *Am. Nat.* **170,** 331–342 (2007).

37. Sexton, D. J. & Schuster, M. Nutrient limitation determines the fitness of cheaters in bacterial siderophore cooperation. *Nat. Commun.* **8,** 230 (2017).

38. Ghoul, M. *et al.* Pyoverdin cheats fail to invade bacterial populations in stationary phase. *J. Evol. Biol.* **29,** 1728–1736 (2016).

39. Gore, J., Youk, H. & van Oudenaarden, A. Snowdrift game dynamics and facultative cheating in yeast. *Nature* **459,** 253–256 (2009).

40. Özkaya, Ö., Xavier, K. B., Dionisio, F. & Balbontín, R. Maintenance of microbial cooperation mediated by public goods in single- and multiple-trait scenarios. *J. Bacteriol.* **199,** e00297-17 (2017).

41. Özkaya, Ö., Balbontín, R., Gordo, I. & Xavier, K. B. Cheating on Cheaters Stabilizes Cooperation in Pseudomonas aeruginosa. *Curr. Biol.* **28,** 2070–2080 (2018).

42. Sandoz, K. M., Mitzimberg, S. M. & Schuster, M. Social cheating in Pseudomonas aeruginosa quorum sensing. *PNAS* **104,** 15876–15881 (2007).

43. Mellbye, B. & Schuster, M. The Sociomicrobiology of Antivirulence Drug Resistance: a Proof of Concept. *MBio* **2,** 3–6 (2011).

44. Gerdt, J. P. & Blackwell, H. E. Competition Studies Confirm Two Major Barriers That Can Preclude the Spread of Resistance to Quorum-Sensing Inhibitors in Bacteria. *ACS Chem. Biol.* **9,** 2291–2299 (2014).

45. Wilder, C. N., Diggle, S. P. & Schuster, M. Cooperation and cheating in Pseudomonas aeruginosa: the roles of the las, rhl and pqs quorum-sensing systems. *ISME J.* **5,** 1332–1343 (2011).

46. Popat, R. *et al.* Quorum-sensing and cheating in bacterial biofilms. *Proc R Soc B* **279,** 4765–4771 (2012).

47. Köhler, T., Perron, G. G., Buckling, A. & van Delden, C. Quorum Sensing Inhibition Selects for Virulence and Cooperation in Pseudomonas aeruginosa. *PLoS Pathog.* **6,** e1000883 (2010).

48. Diggle, S. P., Griffin, A. S., Campbell, G. S. & West, S. A. Cooperation and conflict in quorum-sensing bacterial populations. *Nature* **450,** 411–414 (2007).

49. Allen, R. C., Mcnally, L., Popat, R. & Brown, S. P. Quorum sensing protects bacterial co-operation from exploitation by cheats. *ISME J.* **10,** 1706–1716 (2016).

50. Wang, M., Schaefer, A. L., Dandekar, A. A. & Greenberg, E. P. Quorum sensing and policing of Pseudomonas aeruginosa social cheaters. *PNAS* **112,** 2187–2191 (2015).

51. Greig, D. & Travisano, M. The Prisoner's Dilemma and polymorphism in yeast SUC genes. *Proc. R. Soc. London. Ser. B Biol. Sci.* **271,** S25–S26 (2004).

52. Maclean, R. C., Fuentes-hernandez, A., Greig, D., Hurst, L. D. & Gudelj, I. A Mixture of '"Cheats"' and '"Co-Operators"' Can Enable Maximal Group Benefit. *PLoS Biol.* **8,** e1000486 (2010).

53. Drescher, K., Nadell, C. D., Stone, H. A., Wingreen, N. S. & Bassler, B. L. Solutions to the public goods dilemma in bacterial biofilms. *Curr. Biol.* **24,** 50–55 (2014).

54. Xavier, J. B., Kim, W. & Foster, K. R. A molecular mechanism that stabilizes cooperative secretions in Pseudomonas aeruginosa. *Mol. Microbiol.* **79,** 166–179 (2011).

55. Chao, L. & Levin, B. R. Structured habitats and the evolution of anticompetitor toxins in bacteria. *Proc Natl Acad Sci U S A* **78,** 6324–6328 (1981).

56. Adams, J., Kinney, T., Thompson, S., Rubin, L. & Helling, R. B. Frequency-dependent selection for plasmid-containing cells of Escherichia coli. *Genetics* **91,** 627–637 (1979).

57. Mund, A., Diggle, S. P. & Harrison, F. The Fitness of Pseudomonas aeruginosa Quorum Sensing Signal Cheats Is Influenced by the Diffusivity of the Environment. *MBio* **8,** e00353-17 (2017).

58. Rumbaugh, K. P. *et al.* Quorum Sensing and the Social Evolution of Bacterial Virulence. *Curr. Biol.* **19,** 341–345 (2009).
